# Comparison of static and mineralogical ARD prediction methods in the Nordic environment

**DOI:** 10.1007/s10661-018-7096-2

**Published:** 2018-11-13

**Authors:** Teemu Karlsson, Marja Liisa Räisänen, Marja Lehtonen, Lena Alakangas

**Affiliations:** 1 Industrial Environments and Recycling Unit, Geological Survey of Finland, P.O. Box 1237, 70211 Kuopio, Finland; 2 Department of Civil, Environmental and Natural Resources Engineering, Division of Geosciences and Environmental Engineering, Luleå University of Technology, 97187 Luleå, Sweden; 3 Mineral Processing and Materials Research Unit, Geological Survey of Finland, P.O. Box 96, 02151 Espoo, Finland

**Keywords:** ABA test, NAG test, SEM, Waste rock, Risk assessment

## Abstract

Acid rock drainage (ARD) is a major problem related to the management of mining wastes, especially concerning deposits containing sulphide minerals. Commonly used tests for ARD prediction include acid–base accounting (ABA) tests and the net acid generation (NAG) test. Since drainage quality largely depends on the ratio and quality of acid-producing and neutralising minerals, mineralogical calculations could also be used for ARD prediction. In this study, several Finnish waste rock sites were investigated and the performance of different static ARD test methods was evaluated and compared. At the target mine sites, pyrrhotite was the main mineral contributing to acid production (AP). Silicate minerals were the main contributors to the neutralisation potential (NP) at 60% of the investigated mine sites. Since silicate minerals appear to have a significant role in ARD generation at Finnish mine waste sites, the behaviour of these minerals should be more thoroughly investigated, especially in relation to the acid produced by pyrrhotite oxidation. In general, the NP of silicate minerals appears to be underestimated by laboratory measurements. For example, in the NAG test, the slower-reacting NP-contributing minerals might require a longer time to react than is specified in the currently used method. The results suggest that ARD prediction based on SEM mineralogical calculations is at least as accurate as the commonly used static laboratory methods.

## Introduction

Acid rock drainage (ARD) with high concentrations of potentially harmful elements is considered as one of the main concerns related to the management of mining wastes (e.g. MEND 1991; Price 2003, Dold 2014; Mehta et al. 2018). This particularly applies to deposits containing sulphide minerals, which are prone to oxidisation under the influence of atmospheric conditions (e.g. Singer and Stumm 1970; Blowes and Ptacek 1994). Drainage quality largely depends on the mineralogical and chemical composition of the mine wastes, and particularly on the ratio of acid-producing and neutralising minerals (e.g. Blowes and Jambor 1990; Blowes and Ptacek 1994), combined with reactions catalysed by microbes (e.g. Singer and Stumm 1970). The main acid-producing sulphide species include iron sulphides, such as pyrite (FeS_2_) and pyrrhotite (Fe_1 – *x*_S), whereas the most common neutralising minerals are relatively fast-reacting carbonates (e.g. calcite (CaCO_3_) and dolomite (CaMg(CO_3_)_2_) and slower-reacting silicates, e.g. mica and chlorite (e.g. Ptacek and Blowes 1992; Blowes and Ptacek 1994; Plumlee 1999; Becker et al. 2015). The main sulphide oxidation and acid neutralisation reactions are presented in more detail, for example, by Seal and Hammarstrom (2003) and Dold (2010).

Since ARD plays a major role in the generation of environmental issues, the accurate prediction of ARD is of utmost importance (e.g. Parbhakar-Fox and Lottermoser 2015; Dold 2017). Nevertheless, prediction of the ability of mine waste materials to produce ARD is sometimes challenging, as ARD generation depends on various mineralogical, chemical, hydrological and microbiological factors (e.g. Blowes and Jambor 1990; Nordstrom 2000; Dold 2010; Blowes et al. 2014). The characterisation methods for assessing the acid production potential (APP) and ARD risk can be divided into static and kinetic tests (Lapakko 2002). Static tests are short-term (duration usually measured in hours or days) laboratory analyses. They are relatively low cost (around tens of US$) and usually used for preliminary investigation and screening. Kinetic tests are long-term tests (from several months to years), revealing information on the time scale of drainage events (Lapakko 2002). Kinetic tests are more expensive (from approximately hundreds to thousands of US$) and require site-specific planning.

The most commonly used static APP prediction tests are acid–base accounting (ABA) tests, which include determination of the sulphur/sulphide content of rock material to calculate the acid potential (AP) and determination of the neutralisation potential (NP). The potential risk can then be estimated based on the ratio of the NP and AP (neutralisation potential ratio, NPR) or the subtraction NP-AP (net neutralisation potential, NNP), as is customary in Finland, for instance. It is commonly assumed that mine waste is potentially acid-generating if NPR < 1 or NNP < − 20, in the uncertainty zone if NPR is 1–3 or NNP is between − 20 and + 20, and non-acid-generating if NPR > 3 or NNP > 20 (Sobek et al. 1978; White et al. 1999; Price 2009).

Many variations of the ABA test exist, including the standard ABA originally developed for coal mining (Sobek et al. 1978), the modified ABA (Lawrence et al. 1989; Lawrence and Wang 1997) and “acid addition on the basis of the carbonate content” (EN 15875), which is a recommended standard method within Europe. The Finnish mine waste decree 190/2013 (Finnish Government Decree 2013), which is based on European legislation (EC 2009), states that mine waste can be considered inert if the sulphidic S concentration is ≤ 0.1% or the sulphidic S concentration is ≤ 1% and NPR determined by the standard method EN 15875 is > 3, given that the other requirements mentioned in the decree are also fulfilled. The AP is usually calculated based on the total sulphur content of the sample and expressed as a pyrite equivalent, since it is assumed that pyrite is the most common sulphide mineral and that 4 mol of protons is produced during the oxidation process (Colmer and Hinkle 1947; Singer and Stumm 1970; Nordstrom 2000; Dold 2014). The NP is typically estimated by titrating the sample with acid (Price et al. 1997; White et al. 1999), but it can also be calculated, for example, based on the carbonate carbon content of the rock material (Lawrence and Wang 1997).

The ABA method has some disadvantages. Some carbonate minerals containing iron, particularly siderite (FeCO_3_), do not necessarily contribute to neutralisation (Lawrence and Wang 1997; Haney et al. 2006). The method does not take into account the reactive non-carbonate minerals that may contribute to acid neutralisation, e.g. easily dissolving silicate minerals. Moreover, it does not consider the groups of Fe(III) hydroxides and Fe(II) hydroxide sulphates together with metal chlorides and sulphates as a proton source, although they might be a significant source of acidity (Dold 2017). Furthermore, in the ABA test, the AP is calculated by multiplying the wt% of S by the factor 31.25 based on the hypothesis that 2 mol of protons (released from pyrite oxidation) are neutralised by 1 mol of calcite (e.g. Gard Guide; Verburg et al. 2009). However, at a circumneutral pH, the most common carbonate species is bicarbonate (Appelo and Postma 2010), and the calculation factor should then be 62.5, as two times more calcite is needed to neutralize the same quantity of protons (Dold 2017).

Another commonly used static test for ARD prediction is the net acid generation (NAG) test, which is based on the reaction of a sample with hydrogen peroxide (H_2_O_2_), which accelerates the oxidation of sulphide minerals in the sample. During the test, acid generation and acid neutralisation reactions can occur simultaneously, with the end result representing a direct measurement of the net amount of acid generated by the sample (Miller et al. 1997; Stewart et al. 2006). As the test does not estimate the neutralisation potential (Miller and Jeffery 1995), the use of the net acid production potential (NAPP) together with the NAG pH for more a detailed classification of acid generation is recommended (Morin and Hutt 1999; Räisänen et al. 2010). One of the main advantages of the NAG test is that it does not require separate sulphur determinations. The test is also easier to conduct in a field laboratory than other static tests. As a disadvantage, the test does not give a value for the neutralisation potential, and additional NP measurement is therefore required to calculate the NAPP. Some contradictory predictions have also been noted when NAG test results have been compared with ABA results (Morin and Hutt 1999).

The widely used static ABA and NAG tests have known limitations related to the mineralogy of the sample material (White III et al. 1999; Paktunc 1999b; Jambor 2003; Parbhakar-Fox and Lottermoser 2015; Dold 2017; Parbhakar-Fox et al. 2018). For example, the APP may be overestimated if there are other sulphide- or sulfur-containing minerals than rapidly acid-producing pyrite or pyrrhotite. Conversely, the APP may be underestimated if the waste contains large amounts of easily dissolvable and acid-generating iron sulphate minerals or siderite. The NP may be underestimated if the weathering of silicate minerals is not considered in APP estimations (Lawrence and Scheske 1997; Paktunc 1999b). On the other hand, the NP determined in the laboratory using strong acids will sometimes overestimate silicate mineral reactivity and hence the NP (Lawrence and Scheske 1997; Jambor and Blowes 1998). In addition, static tests do not indicate the source minerals of the NP and AP (Lawrence and Scheske 1997; Paktunc 1999b). Therefore, some mineralogical-based approaches have been proposed, e.g. by Lawrence and Scheske (1997), Parbhakar-Fox and Lottermoser (2015) and Jamieson et al. (2015). As pointed out by Dold (2017), scanning electron microscopy (SEM)-based automated mineral quantification methods have considerably developed in the last decades, increasing the available mineralogical data that could be utilised in ARD prediction.

In this study, we compared the ABA test as presented in the standard EN 15875, the single-addition NAG test as presented in the AMIRA guidebook (Smart et al. 2002), SEM mineralogy-based ARD predictions based on Lawrence and Scheske (1997) and Dold (2017) and actual drainage quality at the target mine sites. For the ABA test, the NP was determined by standard titration and was also calculated based on the carbonate content, as presented by Lawrence and Wang (1997). The AP was calculated with the commonly used factor of 31.25 and the factor of 61.5 proposed by Dold (2017), and the results were compared. Data and samples from several Finnish mine waste sites were utilised, representing a wide range of deposit types.

The objective of this study was to assess the functionality of standard static and mineralogical ARD prediction methods in the Nordic environment and whether static laboratory ARD tests could be substituted in some cases by an ARD prediction based only on SEM mineralogy. Furthermore, the ARD-related properties of various Finnish waste rock sites, including the main minerals responsible for neutralisation and the acid generation potential, are presented.

## Materials and methods

In total, 14 waste rock and 14 corresponding seepage water samples were collected from 10 Finnish mine sites representing various Archaean and Palaeoproterozoic deposits, waste rock types and waste rock facilities of different ages (Table 1). The sampling was conducted during various fieldwork campaigns during the years 2014–2017. The waste rock samples consisted of around 15–20 kg of heterogeneous rock material randomly collected from the surface of the waste rock piles above the seepage points. From the Hitura target site, two sets of waste rock and water samples were taken from the same place: the first in 2014 and the second in 2016. The Kylylahti mine site was first visited for waste rock and water samples in 2014 and thereafter revisited for an additional water and rock sample from the same location in 2017. For this second Kylylahti rock sample, which was clearly more weathered than during the first sampling, only static laboratory tests were conducted, not SEM mineralogy. The Horsmanaho and Siilinjärvi mine sites both had two different waste rock piles, an inactive older one and a new, currently active one, both of which were separately sampled.

**Table 1:** The geology of the ore deposits and a description of waste rocks sampled at the target mine sites

Mine site	Commodity	Deposit type	Waste rocks and sulphides related to the deposit	Target waste rock pile active	Reference
Pampalo	Au	Archaean (2.7 Ga) metamorphic hydrothermal orogenic, gold mainly occurring in quartz veining in the native form, with disseminated sulphide ore minerals	Feldspar porphyry, metavolcanics, metasedimentary rocks and metakomatite, soapstone. Pyrite, pyrrhotite and arsenopyrite	2011–	Sorjonen-Ward (1993), Sorjonen-Ward and Luukkonen (2005), GTK (2017a)
Horsmanaho	Talc, Ni	Palaeoproterozoic (1.9 Ga), mixed hydrothermal deposit, closely associated with a lens of massive serpentinite, consisting of magnesite pods, lenses within talc-magnesite rock and talc-rich schistose soapstone	Serpentinite, soapstone, talc schist, skarn, quartz rock, black schist and micaceous schist. Pentlandite, pyrrhotite, chalcopyrite and pyrite	1982–2004 (old), 2004– (new)	Kuronen and Tuokko (1997)
Kylylahti	Cu, Co, Zn, Ni, Au	Palaeoproterozoic (1.9 Ga) mafic–ultramafic mixed hydrothermal VMS, disseminated sulphide ore hosted by quartz rock and metacarbonate rock	Mica schist, black schist, serpentinite, talc-carbonate rock, carbonate-skarn rock and quartz rock. Pyrite, pyrrhotite, chalcopyrite and sphalerite	2012–	Kontinen et al. (2006)
Hitura	Ni, Co	Palaeoproterozoic (1.9 Ga) magmatic ultramafic intrusion-hosted nickel deposit, consisting of closely spaced serpentinite massifs surrounded by migmatised mica gneiss	Mica gneiss and amphibole rocks (serpentinite in a separate pile). Pyrrhotite, pentlandite, chalcopyrite, vallerite, mackinawite and cubanite	1970–1993	Meriläinen et al. (2008), Makkonen (2012)
Siilinjärvi	Apatite	Archaean (2.6 Ga) metamorphosed glimmerite-carbonatite deposit, intruded by several generations of younger mafic to intermediate dykes and a tonalite–diorite intrusion	Carbonatite, silicocarbonatite, carbonatite–glimmerite, glimmerite, fenites, tonalite–diorite and diabase	1975–ca. 2000 (old), ca. 2000– (new)	O’Brien et al. (2015)
Hammaslahti	Cu, Zn, Au	Palaeoproterozoic (2.2 Ga) siliciclastic–mafic mixed hydrothermal VMS, ore appearing as remobilised breccia, a stringer-like impregnation network and banded breccias, as well as ore rocks with disseminated sulphides	Mica schist, black schist, phyllite, quartz-feldspar schist, dolomite, amphibolite. Chalcopyrite, pyrrhotite, pyrite and sphalerite	1973–1986	Karppanen (1986), Papunen (1986), Pelkonen et al. (1973), Hämäläinen (1987), Loukola-Ruskeeniemi et al. (1993)
Särkiniemi	Ni	Palaeoproterozoic (1.9 Ga) metamorphosed magmatic deposit, the main ore sulphides occur disseminated in the eastern, gabbro-hosted ore body and disseminated, net-textured and massive in the western, peridotite-hosted ore body	Mica gneiss, peridotite, gabbro and hornfels. Pyrrhotite, pentlandite and chalcopyrite	2007–2008	Makkonen and Halkoaho (2007), Kontoniemi and Forss (1997)
Hällinmäki (Virtasalmi)	Cu	Palaeoproterozoic (1.9 Ga) mafic basinal hydrothermal SedEx (sedimentary exchalative) deposit, the ore appearing as brecciated and disseminated in amphibole host rock	Mica- and diopside gneisses, amphibolites, skarn and calcite stone. Chalcopyrite, cubanite, and pyrrhotite, with lesser amounts of pyrite, sphalerite, pentlandite, mackinawite, molybdenite, bornite and other Fe- and Cu-containing sulphides	1966–1984	Hyvärinen (1969), Lawrie (1992), Papunen (1986), GTK (2017b)
Laiva (Laivakangas)	Au	Palaeoproterozoic (1.9 Ga) orogenic metamorphic hydrothermal deposit hosted by silicified shear zones and quartz veins within quartz diorite and intermediate to mafic metavolcanic rocks, cut by post-mineralisation granite	Quartz diorite, mafic volcanic rock, quartz vein and granite. Arsenopyrite	2011–2013	Västi et al. (2012)
Kevitsa	Ni, Cu, PGE	Palaeoproterozoic (2.1 Ga) mafic–ultramafic magmatic deposit hosted within a composite ultramafic layered intrusion, the ore appearing in olivine-pyroxenite as disseminated sulphides	Olivine-pyroxenite, olivine-websterite, gabbro and dunite. Pyrrhotite, pentandite and chalcopyrite	2012–	Santaguida et al. (2015)

The quality assessment of the mine waste drainage included the analysis of pH and alkalinity, which were measured from small seepage streams flowing from the base of the waste rock piles. In two cases (Horsmanaho new pile and Hammaslahti), no visible flowing stream could be found, and the samples were taken from a pond at the waste rock pile. The measurement was conducted at the site using a portable multi-parameter YSI sonde, which was calibrated to pH 4 and 7 prior to every field trip. The alkalinity was determined with a Hach titrator with 0.16 or 1.60 N H_2_SO_4_ to an end point of pH 4.5. The titration pH was measured with the Mettler Toledo SevenGo pH meter, which was calibrated daily to pH 4 and 7.

The mine waste samples were analysed in an accredited laboratory (Labtium Oy). Laboratory duplicates for four waste rock samples and certified reference materials were employed for quality control of the analyses. The rock samples were first dried at < 40 °C and then crushed (> 70%, < 2 mm). For the different chemical and mineralogical analyses, the crushed samples were split with riffles and/or the cone and quartering method and ground in a steel container. The total concentrations of C and S were determined using a pyrolytic method (the Leco method) and IR detection. The amount of carbonate C was calculated based on the difference between total C and non-carbonate C. Total C was determined by pyrolysis and IR detection and non-carbonate C after hydrochloric acid treatment by pyrolysis and IR detection.

### Mineralogical analyses

Mineralogical analyses were conducted at the GTK Research Laboratory by FE-SEM-EDS: JEOL JSM 7100F Schottky, combined with an Oxford Instruments EDS spectrometer X-Max 80 mm^2^ (SDD). The crushed and ground sample materials for geochemical analysis were also used to prepare mineralogical polished samples cast in epoxy and covered with graphite to enhance electric conductivity. To investigate the modal mineralogy, around 10,000 individual mineral particles were analysed from each sample utilising Feature software. The results are semi-quantitative and were normalised to 100%.

Mineral identification was based on comparing the numerical element composition obtained from EDS spectra with the mineralogical database of GTK. For technical reasons, usually around 5–10% of mineral identifications are classed as “unclassified”. This class mainly includes mixed analyses generated from various mineral phases. The amount of unclassified minerals is usually larger when analysing fine particle samples and/or samples with complex mineralogy. Precise identification of minerals based on EDS spectra is not always possible, for instance because carbon and OH and H_2_O groups are not shown in the analysis, which should be taken into account when examining the results.

As a quality control measure for the mineralogical analyses by FE-SEM-EDS, the samples were also analysed by X-ray diffraction (Bruker D8 Discover A25) in order to verify the identification of the main mineral phases. The accuracy of mineral classification based on the GTK mineral database was routinely monitored for each analysed sample. The analytical data that were left unclassified were checked manually.

### NAG test

A single-addition NAG test was performed as proposed by Smart et al. (2002). A mixture of 2.5 g of pulverized sample and 250 ml of 15% (*m*/*V*) H_2_O_2_ was allowed to react for about 12 h, after which the mixture was boiled until the visible reaction ceased. After cooling, pH was measured and the suspension was titrated with NaOH (0.1 mol/L) to pH 4.5 and 7.0. The NAG values were calculated based on the amount of NaOH consumed in the titration and expressed as kg H_2_SO_4_/t (Miller et al. 1997; Smart et al. 2002).

### Determination of AP

The laboratory tests to determine the acid-generating potential of a waste rock sample included the modified ABA test based on the EN 15875 standard (European Committee for standardization 2008). In the ABA test, the AP was calculated by multiplying the wt% of S by the factor 31.25, as proposed in the standard, and by the factor of 62.5 proposed by Dold (2017). The mineralogical AP (minAP) was calculated by multiplying the wt% of S in the mineral with the wt% of the mineral in the sample to obtain the wt% of S in the sample, which was multiplied by 31.25 (EN 15875) or 62.5 (Dold 2017). These factors assume that the oxidation of 1 mol of pyrite via oxygen produces 4 mol of H^+^, and all of the S is assumed to be generated by pyrite, although in mineralogical AP calculations, the amount of H^+^ produced by different sulphide species is also taken into account; chalcopyrite (CuFeS_2_) and pyrrhotite oxidation produce only 2 mol of H^+^, and the factor is therefore divided by 2, while sphalerite ((Zn, Fe)S) and galena (PbS) oxidation produce 1 mol of H^2^, and the factor is therefore 0 (Dold 2017).

### Determination of the NP

The static test to determine the NP by titration was based on the EN 15875 standard (European Committee for standardization 2008), which was originally a modification of the method by Lawrence and Wang (1997). Unlike the modified ABA test by Lawrence and Wang (1997), the standard EN 15875 method does not include the Fizz test to determine the amount of acid needed. Instead, the addition of the acid is based on the total carbon content determined by the EN 13137:2001 standard. A pulverised sample of 2 g was mixed with demineralised water, hydrochloric acid (1.0 mol/L) was added and the mixture was stirred until pH 2.0–2.5 was reached after 24 h. If the pH was above 2.5 at 22 h, more HCl was added to adjust the pH between 2.0 and 2.5 and preferably as close to 2.0 as possible. After the stabilisation of pH, the solution was titrated with sodium hydroxide (0.1 mol/L) to an end point pH of 8.3. The volume of NaOH indicates how much of the HCl the sample has consumed, therefore reflecting the neutralisation potential, which was calculated as CaCO_3_ equivalents in kilograms per ton.

The carbonate NP (carbNP) value was calculated on the basis of the carbonate C concentration, also yielding the value in units of kg CaCO_3_/t. The carbC value was multiplied by a factor of 83.3, which was calculated as follows:

$$ {100.06}_{\left(\mathrm{mole}\ \mathrm{weight}\ \mathrm{calcite}\right)}/{12.01}_{\left(\mathrm{mole}\ \mathrm{weight}\ \mathrm{carbon}\right)}\times {10}_{\left(\mathrm{tCaCO}3/1000\mathrm{t}\right)}=83.3 $$

Mineralogical NP (minNP) values were determined by calculations based on mineralogy as presented by Dold (2017) and Lawrence and Scheske (1997), respectively. As calcite is considered to be the most important neutralising mineral in the mining environment, it is commonly considered in calculations for the NP and results are expressed as its equivalent (e.g. Dold 2017). The mineralogical NP for a carbonate mineral converted to X kg CaCO_3_/t was calculated by dividing the mole weight of carbon by the total mole weight of the carbonate mineral and then multiplying by the mineral concentration (wt%) with the factor 83.3. For example, the minNP for 2 wt% of magnesite (MgCO_3_) was calculated as follows:

$$ {\mathrm{minNP}}_{\left(\mathrm{magnesite}\right)}={12.01}_{\left(\mathrm{mole}\ \mathrm{weight}\ \mathrm{carbon}\ \mathrm{g}/\mathrm{mol}\right)}/{84.31}_{\left(\mathrm{mole}\ \mathrm{weight}\ \mathrm{magnesite}\ \mathrm{g}/\mathrm{mol}\right)}\times {2}_{\left(\mathrm{magnesite}\ \mathrm{wt}.\%\right)}\times 83.3=23.74{\mathrm{kgCaCO}}_3/\mathrm{t}. $$

Mineralogical NP calculations for non-carbonate minerals were performed according to Lawrence and Scheske (1997) by adding together the weighted NP values for each (significant) mineral present in the sample. To simplify the minNP calculations, non-carbonate minerals classified into slow-weathering, very slow-weathering and inert groups were not taken into account, as their NP contribution is insignificant (Sverdrup 1990; Kwong 1993). In addition to carbonate minerals, only the silicate minerals classified as fast- and intermediate-weathering by Sverdrup (1990) with a total mass % above 1 were included in the calculations. In contrast to the Cross, Iddings, Pirsson and Washington (CIPW) normative mineralogical composition applied by Lawrence and Scheske (1997), which is not well suited to metamorphosed rocks (Paktunc 1999a), mineralogical compositions for the minNP calculations were obtained by SEM-based automated mineral quantification, as described above.

The relative reactivity values applied in this study were obtained from the original references (Sverdrup 1990, Kwong 1993). Sverdrup (1990) stated that the relative reactivity of minerals changes depending on the total percentages of different mineral groups in the sample (Table 2). To simplify the calculations in this study, the relative reactivity of the average mineral class content of 30% was used, as this is probably close to the average realistic mineral class contents in a typical rock. Calculated minNP values were converted to kg CaCO_3_ by using the molecular weight of CaCO_3_ for the mineral. The selected minerals and their molecular weights used in this study are presented in Table 3.

**Table 2:** Relative reactivity in terms of the acid neutralisation capacity for selected minerals after Sverdrup (1990) and Kwong (1993)

Mineral class	Typical minerals	Relative reactivity^a^ Average mineral class content			
		100%	30%	3%	0.3%
Carbonates	Calcite, dolomite, magnesite, aragonite, brucite	1	1	1	1
Fast-weathering	Anorthite, olivine, garnet, diopside, wollastonite, jadeite, nepheline, leucite, spodumene	0.6	0.67	0.3	0.1
Intermediate-weathering	Enstatite, augite, hornblende, tremolite, actinolite, biotite, chlorite, serpentine, talc, epidote, zoisite, hedenbergite, glaucophane, anthophyllite, phlogopite^b^, anthophyllite^c^	0.4	0.2	0.03	0.01
Slow-weathering	Plagioclase (Ab100-Ab30), kaolinite, vermiculite, montmorillonite, gibbsite	0.02	0.013	0.002	–
Very slow-weathering	K-feldspar, muscovite	0.01	0.007	0.001	–
Inert	Quartz, rutile, zircon	0.004	0.0007	–	–

In this study, the relative reactivity of the average mineral class content of 30% was used

^a^In soil, at pH 5

^b^Phlogopite added to the intermediate-weathering class based on Schweda and Kalinowski (1994)

^c^Anthophyllite added to the intermediate-weathering class based on Chen and Brantley (1998) and Rozalen et al. (2014)

**Table 3:** Formulas and molecular weights of selected minerals used in this study (Mineralogy Database 2017)

Mineral	Formula	Mol. wt. (g/mol)
Carbonates		
Calcite	CaCO_3_	100.09
Dolomite	CaMg(CO_3_)_2_	184.4
Magnesite	MgCO_3_	84.31
Silicates		
Actinolite	Ca_2_(Mg,Fe)_5_Si_8_O_22_(OH)_2_	853.16
Aegerine-augite	(Ca,Na)(Mg,Fe)[Si_2_O_6_]	228.05
Almandine	Fe_3_Al_2_(SiO_4_)_3_	497.75
Anorthite	CaAl_2_Si_2_O_8_	277.41
Anthophyllite	Mg_7_(Si_8_O_22_)(OH)_2_	780.82
Augite	(Ca,Na)(Mg,Fe,Al,Ti)(Si,Al)_2_O_6_	236.35
Biotite	K(Mg,Fe)_3_(AlSi_3_O_10_)(OH,F)_2_	433.53
Chlorite^a^	(Mg,Fe)_5_Al(Si_3_Al)O_10_(OH)_8_	595.22
Chrysotile	Mg_3_Si_2_O_5_(OH)_4_	277.11
Diopside	CaMgSi_2_O_6_	216.55
Epidote	Ca_2_Al_2_(Fe^3+^;Al)(SiO_4_)(Si_2_O_7_)O(OH)	519.3
Fe-hornblende	Ca_2_[Fe_4_(Al,Fe)]Si_7_AlO_22_(OH)_2_	947.32
Mg-hornblende	Ca_2_[Mg_4_(Al,Fe)]Si_7_AlO_22_(OH)_2_	821.16
Olivine	(Mg,Fe)_2_SiO_4_	153.31
Phlogopite	KMg_3_AlSi_3_O_10_F(OH)	419.25
Serpentine	(Mg,Fe)_3_Si_2_O_5_(OH)_4_	300.77
Talc	Mg_3_Si_4_O_10_(OH)_2_	379.27
Tremolite	Ca_2_Mg_5_Si_8_O_22_(OH)_2_	812.37
Sulphides		
Chalcopyrite	CuFeS_2_	183.53
Pentlandite	(Fe,Ni)_9_S_8_	771.94
Pyrite	FeS_2_	119.98
Pyrrhotite	Fe_1-x_S	85.12
Sphalerite	(Zn,Fe)S	96.98

^a^Here, it is assumed that all chlorite is clinochlore

For example, the NP contribution in kg CaCO_3_ equivalent/t for 30.0 wt% of diopside (CaMgSi_2_O_6_) in a sample would be calculated as follows:

$$ {\displaystyle \begin{array}{l}{\mathrm{minNP}}_{\left(\mathrm{diopside}\right)}=\left(30.0/100\right)\times \left(1000\  kg/1\ \mathrm{tonne}\right)\times \left({100.06}_{\left(\mathrm{mole}\mathrm{weight}\ \mathrm{calcite}\right)}/{216.55}_{\left(\mathrm{mole}\ \mathrm{weight}\ \mathrm{diopside}\right)}\right)\times 0.67=92.9\\ {}\mathrm{kg}\ {\mathrm{CaCO}}_3/\mathrm{t}\end{array}} $$

An example of the mineralogical calculation of the NNP and NPR for a hypothetical sample is presented in Table 4.

**Table 4:** Example calculation for the mineralogical NNP and NPR

Mineral	% total mass	Mineral	wt% S in mineral	wt% of mineral in sample	wt% S in sample	minAP1 (31.25)	minAP2 (62.5)
Plagioclase	35.0	Pyrrhotite	37.67^a^	0.50	0.19	2.94^b^	5.89
Quartz	25.0	Pyrite	53.30	0.30	0.16	5.00^c^	9.99
Biotite	20.0	Total minAP				7.94	15.88
Mg-Hornblende	6.0		wt% C in mineral	wt% of mineral in sample	wt% C in sample	minNP	
K-feldspar	6.0	Calcite	12.00	0.20	0.02	1.67^d^	
Fe-Hornblende	4.0	Biotite		20.00		61.94^e^	
Serpentine	3.0	Mg-Hornblende		6.00		4.90^f^	
Pyrrhotite	0.5	Fe-Hornblende		4.00		2.83^g^	
Pyrite	0.3	Serpentine		3.00		6.69^h^	
Calcite	0.2	Total minNP				**78.03**	
Total	100.0						
					NNP	70.09	62.15
					NPR	9.83	4.91

^a^wt.% S in mineral based on the formula Fe_0.95_S for pyrrhotite

^b^0.19 (wt% S) × 31.25/2, as the oxidation of pyrrhotite via oxygen produces only half the amount of H^+^ compared to pyrite

^c^0.16 (wt% S) × 31.25

^d^0.02 (wt% C) × 83.3

^e^20 (wt%) / 100 × 1000 kg/t × 100.09 (g/mol) / 216.55 (g/mol) × 0.67

^f^6 (wt%) / 100 × 1000 kg/t × 100.09 (g/mol) / 821.16 (g/mol) × 0.67

^g^4 (wt%) / 100 × 1000 kg/t × 100.09 (g/mol) / 947.32 (g/mol) × 0.67

^h^3 (wt%) / 100 × 1000 kg/t × 100.09 (g/mol) / 300.77 (g/mol) × 0.67

## Results

Measured seepage water pHs and alkalinities are presented in Table 5. Modal mineral contents based on EDS spectra are presented in Table 6. Minerals that occur in larger amounts than 1% are included, as well as important minerals related to the AP and NP (sulphides and carbonates, respectively) in lesser amounts. The samples included various minerals that could not be precisely identified by EDS analysis, e.g. different Mg silicates such as serpentine ((Mg,Fe)_3_Si_2_O_5_(OH)_4_), Mg amphiboles and Mg pyroxenes. Therefore, the classification of amphiboles and pyroxenes is indicative, and the division between tremolite (Ca_2_Mg_5_Si_8_O_22_(OH)_2_) and diopside is uncertain. Although FE-SEM-EDS is not suitable for the identification of minerals containing OH and H_2_O groups, some small amounts (0.01–0.02 wt%) of gypsum (CaSO_4_ · 2H_2_O) and silicate-gypsum mixture were detected in several samples.

**Table 5:** Seepage water pH and alkalinity measured at mine waste sites

Mine site/sample	pH	Alkalinity mg/L CaCO_3_
Pampalo	6.7	239
Siilinjärvi old	6.5	526
Siilinjärvi new	7.0	134
Horsmanaho old	7.3	87
Horsmanaho new	7.7	610
Kylylahti 2014	7.1	188
Kylylahti 2017	2.9	–
Hitura 2014	3.5	–
Hitura 2016	4.0	–
Hammaslahti	3.9	–
Särkiniemi	3.3	–
Hällinmäki	6.7	8
Laiva	7.0	21
Kevitsa	7.4	100

**Table 6:** Mineralogy of the waste rock samples based on FE-SEM-EDS

Mineral	Pampalo	Siilinjärvi old	Siilinjärvi new	Horsmanaho old	Horsmanaho new	Kylylahti 2014	Hitura 2014	Hitura 2016	Hammaslahti	Särkiniemi	Hällinmäki	Laiva	Kevitsa
Calcite	0.09	18.9	4.41	0.39	0.08	1.89			0.02		0.06		0.04
Dolomite	4.21	7.27		3.34	2.88	0.02	0.01						
Magnesite				9.04	9.97								
Siderite	0.14												
Carbonates (sum)	4.44	26.17	4.41	12.77	12.93	1.91	0.01		0.02		0.06		0.04
Anorthite (Ca)											1.2		
Olivine							1.9						2.7
Almandine													
Diopside													47.9
Fast-weathering silicates (sum)							1.9				1.2		50.7
Augite											3.2		
Aegerine-augite			14.4										
Mg-cummingtonite/enstatite										1.1			
Fe-hornblende	2.1	13.5								6.1	8.4	5.9	1.2
Mg-hornblende	1.1								1.5		14.0	2.4	3.5
Tremolite				4.5	6.2	4.9	1.3		1.4				12.7
Actinolite		2.2							1.3		3.4	7.9	2.4
Biotite	18.9	32.8	14.6	1.4	10.7	14.5	19.5	25.4	6.4	33.6	1.2	10.9	
Chlorite	3.0			13.6			1.0		8.3	1.4			1.3
Serpentine				1.5			10.1			3.5			9.8
Talc	4.4			6.5	5.0								
Epidote											2.8		
Clinozoisite		2.6										1.8	
Anthophyllite													3.0
Phlogopite		8.0				7.3							
Skapolite													3.6
Apatite		3.1											
Titanite			1.5										
Ilmenite				1.2									
Intermediate-weathering silicates (sum)	29.6	62.1	30.5	28.7	21.9	26.7	31.9	25.4	18.9	45.6	33.0	28.8	37.4
Plagioclase (Na,Ca)	1.1			9.1	6.5	24.1	8.3	18	1.9	19.7	42.2	21.5	2.4
Albite (Na)	28.6	3.5	47.1		1.5	3.8	8.1	16.4	6.6	1.5	1.7	10.8	
Slow-weathering silicates	29.7	3.5	47.1	9.1	8.0	27.9	16.4	34.4	8.5	21.2	43.8	32.3	2.4
K-feldspar	15.6		15.6	2.4	1.5				2.2			13.8	
Muscovite	4.1					4.1	5.6	7.0	3.8				
Very slow-weathering silicates	19.7		15.6	2.4	1.5	4.1	5.6	7.0	6.0			13.8	
Inert silicates: quartz	11.2	1.0		38.7	45.3	16.4	20.6	14.2	42.3	16.6	5.0	20.3	
Pyrite			0.22	0.77	0.63	8.79	1.11	0.1	0.06	0.02	0.01		
Pyrrhotite	0.01	0.01	0.12	1.13	3.59	0.61	7.54	1.83	0.83	1.52	0.11	0.1	0.15
Chalcopyrite	0.03						0.82	0.07	0.08		0.16		0.03
Pentlandite				0.11	0.59	0.05				0.03			0.05
Sphalerite						0.08	0.01	0.01					
Mix of sulphides and silicates								5.8^a^		2.54^b^			
Sulphides (sum)	0.04	0.01	0.34	2.01	4.81	9.53	9.48	4.33	0.97	3.81	0.28	0.1	0.23
Oxidized Fe sulphide			0.01	0.08	0.04	0.02	0.94	0.23	0.49	0.14			
Unclassified minerals	3.0	2.8	3.5	5.3	2.6	11.8	9.6	6.8	19.7	7.6	5.8	3.5	6.4

Minerals with contents of > 1 wt% are presented, as well as sulphides and carbonates with contents of < 1 wt%. The minerals are grouped according to their weathering intensity after Sverdrup (1990)

^a^Mixture of around 3/5 unclassified silicates and 2/5 sulphides; 2.32 wt% added to pyrrhotite in mineralogical AP calculations. Estimation based on SEM raw data

^b^Mixture of around 1/2 unclassified silicates and 1/2 sulphides; 1.27 wt% added to pyrrhotite in mineralogical AP calculations. Estimation based on SEM raw data

Geochemical analysis results are presented in Table 7, together with different APs and NPs calculated using various methods. According to the total S and carbonate C concentrations, the waste rock samples can be classified into three groups: samples of group I have minor sulphide sulphur, are close to inert (≤ 0.1 wt% S) and have high or moderate carbonate C contents; samples of group II have a relatively high sulphide sulfur content and high to moderate carbonate C contents; and samples of group III have variable contents of sulphide sulphur and low or no carbonate carbon contents (≤ 0.1 wt% carbC). The contributions of different minerals to the total minNP of the samples are presented in Table 8. From the calculated AP and NP values, different NNPs and NPRs were derived with an AP factor of 31.25 (Table 9) and an AP factor of 62.5 (Table 10).

**Table 7:** Geochemical analysis results together with AP and NP values calculated using various methods

Sample group/mine site	S %	minS %	C %	carbC %	NAG (pH 4.5) kg H_2_SO_4_/t	NAG (pH 7.0) kg H_2_SO4/t	NAGpH pH	AP1^a^ kg CaCO3/t	AP2^b^ kg CaCO3/t	minAP1^c^ kg CaCO3/t	minAP2^d^ kg CaCO3/t	NP kg CaCO3/t	carbNP^e^ kg CaCO3/t	minNP^f^ kg CaCO3/t
Group I														
Pampalo	0.1 ± 0.1	0.007	0.85 ± 0	0.82 ± 0.01	0 ± 0	0 ± 0	9.2 ± 0.1	3.13	6.25	0.22	0.44	71.5 ± 0.1	68.3	59.4
Siilinjärvi old	0.07	0.004	2.82	2.77	0	0	10.8	2.19	4.38	0.06	0.12	259	231	290
Siilinjärvi new	0.17	0.17	0.80	0.76	0	0	10.5	5.31	10.6	4.37	8.74	113	63.3	63.5
Group II														
Horsmanaho old	1.41	0.88	2.10	1.32	0	0	7.5	44.1	88.1	20.1	40.2	41.7	110	158
Horsmanaho new	2.27	1.89	3.19	1.35	2.62	7.39	3.8	70.9	142	34.7	69.4	46.3	113	160
Kylylahti 2014	4.32 ± 0.01	4.97	4.19 ± 0	0.65 ± 0	0 ± 0	0 ± 0	9.0 ± 0.2	135	270	150	301	62.2 ± 0.1	54.2	28.5
Group III														
Hitura 2014	3.35	3.72	0.91	0.11	22.1	6.92	2.6	105	209	67.3	135	38.3	9.17	24.8
Särkiniemi	1.69	1.07	0.26	0.05	18.4	30.3	2.7	52.8	106	16.9	33.8	13.2	4.48	19.6
Kevitsa	0.31	0.09	0.15	0.1	0	0	9.1	9.56	19.1	1.31	2.61	57.7	8.1	173
Hammaslahti	1.61 ± 0.01	0.37	0.53 ± 0.01	<0.05	8.35 ± 0	18.6 ± 0	3.0 ± 0.04	50.3	101	6.32	12.6	13.4 ± 0	0	6.97
Hitura 2016	2.47	1.63	1.04	<0.05	25.1	36.7	2.6	77.2	154	26.5	53.0	4.55	0	13.6
Hällinmäki	0.37	0.11	<0.05	<0.05	0.24	2.63	4.1	11.5	22.9	1.69	3.37	9.52	0	20.3
Laiva	0.1	0.04	<0.05	<0.05	0	0	7.5	3.16	6.31	0.06	0.12	9.91	0	8.7
Kylylahti 2017	10.1 ± 0	–	0.08 ± 0.02	<0.05	23.4 ± 0.4	56.8 ± 0.1	2.6 ± 0.02	315.6	631.3	–	–	6.4 ± 0.4	0	–

Duplicate analysis was performed for four samples in the laboratory; these values are presented as averages of the two measurements ± standard deviation (*n* = 2)

^a^AP1 = 31.25 × S wt%

^b^AP2 = 62.5 × S wt%

^c^minAP1 calculated with the factor of 31.25 and considering the sulphide species

^d^minAP2 calculated with the factor of 62.5 and considering the sulphide species

^e^Neutralisation potential = 83.3 × CarbC

^f^minNP = calculated as explained above

**Table 8:**
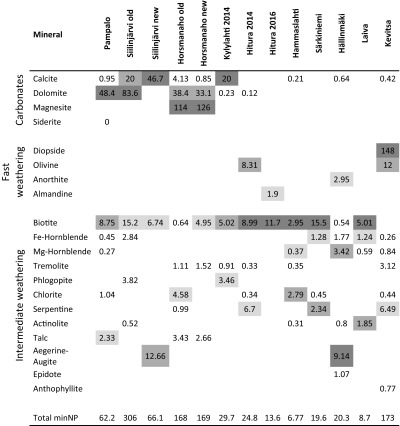
Contributions of different minerals to the total minNP of the samples

Values as kg CaCO_3_/t. Highest contribution to NP is presented in dark grey, second highest contribution to NP in medium grey and third highest contribution to NP in light grey

**Table 9:**
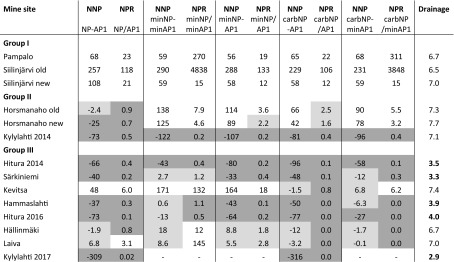
NNP and NPR values calculated with different APs and NPs and compared with the drainage pH

AP1 factor = 31.25. Non-acid-generating is presented in white cells, potentially acid-generating in dark grey and uncertainty zone in light grey

**Table 10:**
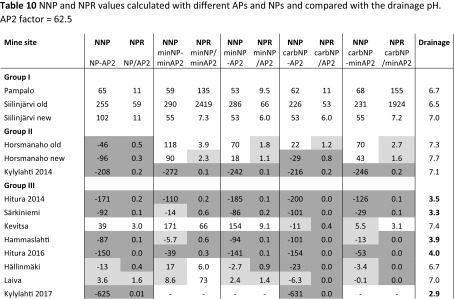
NNP and NPR values calculated with different APs and NPs and compared with the drainage pH

AP2 factor = 62.5. Non-acid-generating is presented in white cells, potentially acid-generating in dark grey and uncertainty zone in light grey

The main results are presented below according to the target mine site, after which some general observations are summarised.

### Pampalo

Drainage water from the Pampalo waste rock pile had a neutral pH (6.7) and high alkalinity (239 mg/L CaCO_3_). The main minerals (wt% > 5) were albite (NaAlSi_3_O_8_, 28.6 wt.%), biotite (K(Mg,Fe)_3_(AlSi_3_O_10_)(OH,F)_2_, 18.9 wt%), k-feldspar (KAlSi_3_O_8_, 15.6 wt.%) and quartz (SiO_2_, 11.2 wt%). The carbonate mineral content was relatively high (4.44 wt%), the sample containing 4.21 wt% of dolomite, 0.14 wt% of siderite and 0.09 wt% of calcite. The total C concentration was 0.85% and the carbonate C concentration 0.82%. The sulphidic mineral content was low (0.04 wt%), the sample containing 0.03 wt% of chalcopyrite and 0.01 wt% of pyrrhotite. The sulphides appeared as single and unweathered, mainly < 10 μm grains. No oxidised Fe sulphides were detected. The total S concentration was 0.1% based on the Leco method and 0.007% according to calculation based on the sulphide mineral amounts. Based on the S and C concentrations, the Pampalo sample was classified into group I.

The NAG pH of the Pampalo sample was 9.2, both NAG values (pH 4.5 and pH 7.0) being 0 kg H_2_SO_4_/t. The AP calculated based on the total S and applying the factor of 31.25 was 3.13 kg CaCO_3_/t (AP1) and 6.25 kg CaCO_3_/t applying the factor of 62.5 (AP2). The minAP calculated based on minS was 0.22 kg CaCO_3_/t with the factor of 31.25 (minAP1) and 0.44 kg CaCO_3_/t with the factor of 62.5 (minAP2). The NP determined with the standard ABA test was 71.5 kg CaCO_3_/t, the carbNP calculated from the carbonate C concentration was 68.3 kg CaCO_3_/t and the minNP calculated by mineralogy was 59.4 kg CaCO_3_/t. Based on the calculations, the main three minNP-contributing minerals were dolomite (48.4 kg CaCO_3_/t), biotite (8.75 kg CaCO_3_/t) and talc (2.33 kg CaCO_3_/t). The Pampalo waste rock material was classified as non-acid-generating by all the applied ARD prediction methods.

### Siilinjärvi

Drainage water from the Siilinjärvi new waste rock pile had a neutral pH (7.0) and high alkalinity (134 mg/L CaCO_3_). The main minerals were albite (47.1 wt%), k-feldspar (15.6 wt%), biotite (14.6 wt%) and aegerine-augite ((Ca,Na)(Mg,Fe)(Si_2_O_6_), 14.4 wt%). The carbonate mineral content was relatively high (4.41 wt%), the sample containing 4.41 wt% of calcite. The total C concentration was 0.80% and the carbonate C concentration 0.76%. The sulphidic mineral content was low (0.34 wt%), the sample containing 0.22 wt% of pyrite and 0.12 wt% of pyrrhotite. The sulphides appeared as single grains, the maximum diameters being a few hundred micrometres but mainly some tens of micrometres. The appearance of the sulphide grains varied, some being unweathered and some oxidised. The amount of detected oxidised Fe sulphides was 0.01 wt%. The total S concentration was 0.17% according to the Leco method and 0.17% according to calculation based on the sulphide mineral amounts. Based on the S and C concentrations, the Siilinjärvi new sample was classified into group I.

The NAG pH of the Siilinjärvi new sample was 10.5, both NAG values (pH 4.5 and pH 7.0) being 0 kg H_2_SO_4_/t. The AP calculated based on the total S and applying the factor of 31.25 was 5.31 kg CaCO_3_/t (AP1) and 10.6 kg CaCO_3_/t with the factor of 62.5 (AP2). The minAP calculated based on minS was 4.37 kg CaCO_3_/t applying the factor of 31.25 (minAP1) and 8.74 kg CaCO_3_/t with the factor of 62.5 (minAP2). The NP according to the standard ABA test was 113 kg CaCO_3_/t, the carbNP calculated from the carbonate C concentration was 63.3 kg CaCO_3_/t and the minNP calculated by mineralogy was 63.5 kg CaCO_3_/t. Based on the calculations, the main three minNP-contributing minerals were calcite (46.7 kg CaCO_3_/t), aegerine-augite (12.66 kg CaCO_3_/t) and biotite (6.74 kg CaCO_3_/t). The Pampalo waste rock material was classified as non-acid-generating by all the applied ARD prediction methods.

Drainage water from the Siilinjärvi old waste rock pile had a lower pH (6.5) than that from the Siilinjärvi new pile but higher alkalinity (526 mg/L CaCO_3_). The main minerals were biotite (32.8 wt%), calcite (18.9 wt%), Fe-hornblende (Ca_2_[Fe_4_(Al,Fe)]Si_7_AlO_22_(OH)_2_, 13.5 wt%), phlogopite (KMg_3_AlSi_3_O_10_F(OH), 8.0 wt%) and dolomite (7.3 wt%). The carbonate mineral content was high (26.17 wt%). The total C concentration was 2.82% and the carbonate C concentration 2.77%. The sulphidic mineral content was low (0.01 wt%), the sample containing 0.01 wt% of pyrrhotite. The sulphides appeared as single grains and as inclusions inside silicates. The sulphide grains were unweathered and small, 20–30 μm at maximum. No oxidised Fe sulphides were detected. The total S concentration was 0.07% according to the Leco method and 0.004% according to calculation based on the sulphide mineral amounts. Based on the S and C concentrations, the Siilinjärvi old sample was classified into group I.

The NAG pH of the Siilinjärvi old sample was 10.8, both NAG values (pH 4.5 and pH 7.0) being 0 kg H_2_SO_4_/t. The AP calculated based on the total S and applying the factor of 31.25 was 2.19 kg CaCO_3_/t (AP1) and 4.38 kg CaCO_3_/t with the factor of 62.5 (AP2). The minAP calculated based on minS was 0.06 kg CaCO_3_/t applying the factor of 31.25 (minAP1) and 0.12 kg CaCO_3_/t with the factor of 62.5 (minAP2). The NP according to the standard ABA test delivered was 259 kg CaCO_3_/t, the carbNP calculated from the carbonate C concentration was 231 kg CaCO_3_/t and the minNP calculated by mineralogy was 290 kg CaCO_3_/t. Based on the calculations, the main three minNP-contributing minerals were dolomite (83.6 kg CaCO_3_/t), calcite (20.0 kg CaCO_3_/t) and biotite (15.2 kg CaCO_3_/t). The Pampalo waste rock material was classified as non-acid-generating by all the applied ARD prediction methods.

### Horsmanaho

Drainage water from the Horsmanaho new waste rock pile had a neutral pH (7.7) and high alkalinity (610 mg/L CaCO_3_). The main minerals were quartz (45.3 wt%), biotite (10.7 wt%), magnesite (9.97 wt%), plagioclase (other than albite, 6.5 wt%), tremolite (6.2 wt%) and talc (Mg_3_Si_4_O_10_(OH)_2_, 5.0 wt%). The carbonate mineral content was relatively high (12.93 wt.%), the sample containing 9.97 wt% of magnesite, 3.34 wt% of dolomite and 0.39 wt% of calcite. The total C concentration was 3.19% and the carbonate C concentration 1.35%. The sulphidic mineral content was 4.81 wt%, the sample containing 3.59 wt% of pyrrhotite, 0.63 wt% of pyrite and 0.59 wt% of pentlandite ((Fe, Ni)_9_S_8_). The sulphides commonly appeared as inclusions inside silicates. The sulphide grains were mainly unweathered and small, primarily in the size fraction of 10–20 μm. The amount of detected oxidised Fe sulphides was 0.04 wt%. The total S concentration was 2.27% according to the Leco method and 1.89% according to calculation based on the sulphide mineral amounts. Based on the S and C concentrations, the Horsmanaho new sample was classified into group II.

The NAG pH of the Horsmanaho new sample was 3.8, and the NAG values were 2.62 kg H_2_SO_4_/t (pH 4.5) and 7.39 kg H_2_SO_4_/t (pH 7.0). The AP calculated based on the total S and applying the factor of 31.25 was 70.9 kg CaCO_3_/t (AP1) and 142 kg CaCO_3_/t with the factor of 62.5 (AP2). The minAP calculated based on minS was 34.7 kg CaCO_3_/t applying the factor of 31.25 (minAP1) and 69.4 kg CaCO_3_/t with the factor of 62.5 (minAP2). The NP according to the standard ABA test was 46.3 kg CaCO_3_/t, the carbNP calculated from the carbonate C concentration was 113 kg CaCO_3_/t and the minNP calculated by mineralogy was 160 kg CaCO_3_/t. Based on the calculations, the main three minNP-contributing minerals were magnesite (126 kg CaCO_3_/t), dolomite (33.1 kg CaCO_3_/t) and biotite (4.95 kg CaCO_3_/t). The Horsmanaho new waste rock material was classified as non-acid-generating by most of the applied ARD prediction methods.

Drainage water from the Horsmanaho old waste rock pile had a lower pH (7.3) than that from the Horsmanaho new pile and lower alkalinity (87 mg/L CaCO_3_). The main minerals were quartz (38.7 wt%), chlorite ((Mg,Fe)_5_Al(Si_3_Al)O_10_(OH)_8_, 13.6 wt%), plagioclase (other than albite, 9.1 wt%), magnesite (9.04 wt%) and talc (6.5 wt%). Some traces of gypsum were detected (silicate-gypsum mixture 0.02 wt%). The carbonate mineral content was high (12.77 wt%). The total C concentration was 2.10% and the carbonate C concentration 1.32%. The sulphidic mineral content was 2.01 wt%, the sample containing 1.13 wt% of pyrrhotite, 0.77 wt% of pyrite and 0.11 wt% of pentlandite. The sulphides appeared as single grains, primarily in the size fraction of 10–20 μm. The appearance of the sulphide grains varied, some being unweathered and some strongly oxidised. The amount of detected oxidised Fe sulphides was 0.08 wt%. The total S concentration was 1.41% according to the Leco method and 0.88% according to calculation based on the sulphide mineral amounts. Based on the S and C concentrations, the Horsmanaho old sample was classified into group II.

The NAG pH of the Horsmanaho old sample was 7.5, both NAG values (pH 4.5 and pH 7.0) being 0 kg H_2_SO_4_/t. The AP calculated based on the total S and applying the factor of 31.25 was 44.1 kg CaCO_3_/t (AP1) and 88.1 kg CaCO_3_/t with the factor of 62.5 (AP2). The minAP calculated based on minS was 20.1 kg CaCO_3_/t applying the factor of 31.25 (minAP1) and 40.2 kg CaCO_3_/t with the factor of 62.5 (minAP2). The NP according to the standard ABA test was 41.7 kg CaCO_3_/t, the carbNP calculated from the carbonate C concentration was 110 kg CaCO_3_/t and the minNP calculated by mineralogy was 158 kg CaCO_3_/t. Based on the calculations, the main three minNP-contributing minerals were magnesite (114 kg CaCO_3_/t), dolomite (38.4 kg CaCO_3_/t) and chlorite (4.58 kg CaCO_3_/t). The Horsmanaho old waste rock material was classified as non-acid-generating by most of the applied ARD prediction methods.

### Kylylahti

Drainage water from the Kylylahti waste rock pile had a neutral pH (7.1) and high alkalinity (188 mg/L CaCO_3_) in 2014. When the drainage was measured again in 2017, the pH had dropped to 2.9 and the alkalinity could not be measured. The main minerals of the Kylylahti 2014 sample were plagioclase (other than albite, 24.1 wt%), quartz (16.4 wt%), biotite (14.5 wt%), phlogopite (7.3 wt%) and pyrite (8.79 wt%). Some traces of gypsum were detected (silicate-gypsum mixture 0.01 wt%). The Kylylahti 2014 sample contained a substantial amount of unclassified minerals (11.8 wt%). The carbonate mineral content was 1.91 wt%, the sample containing 1.89 wt% of calcite and 0.02 wt% of dolomite. The total C concentration was 4.19% and the carbonate C concentration 0.65%. The sulphidic mineral content was 9.53 wt%, the sample containing 8.79 wt% of pyrite, 0.61 wt% of pyrrhotite, 0.08 wt% of sphalerite and 0.05 wt% of pentlandite. The sulphides appeared as single grains, pyrite primarily in the size fraction of < 20 μm and pyrrhotite in the size fraction of 10–40 μm. The sulphide grains were mainly unweathered, but some grains were slightly or some strongly oxidised. The amount of detected oxidised Fe sulphides was 0.02 wt%. The total S concentration was 4.32% according to the Leco method and 4.97% according to calculation based on the sulphide mineral amounts. Based on the S and C concentrations, the Kylylahti 2014 sample was classified into group II.

The NAG pH of the Kylylahti 2014 sample was 9.0, both NAG values (pH 4.5 and pH 7.0) being 0 kg H_2_SO_4_/t. The AP calculated based on the total S and applying the factor of 31.25 was 135 kg CaCO_3_/t (AP1) and 270 kg CaCO_3_/t with the factor of 62.5 (AP2). The minAP calculated based on minS was 150 kg CaCO_3_/t applying the factor of 31.25 (minAP1) and 301 kg CaCO_3_/t with the factor of 62.5 (minAP2). The NP according to the standard ABA test was 62.2 kg CaCO_3_/t, the carbNP calculated from the carbonate C concentration was 54.2 kg CaCO_3_/t and the minNP calculated by mineralogy was 28.5 kg CaCO_3_/t. Based on the calculations, the main three minNP-contributing minerals were calcite (20 kg CaCO_3_/t), biotite (5.02 kg CaCO_3_/t) and phlogopite (3.46 kg CaCO_3_/t). The Kylylahti 2014 waste rock material was classified as acid-generating by all the applied ARD prediction methods, except NAG pH, which predicted non-acid generation.

### Hitura

In 2014, drainage water from the Hitura waste rock pile had an acid pH (3.5) and the alkalinity could not be measured. In the Hitura 2014 sample, the main minerals were quartz (20.6 wt%), biotite (19.5 wt%), serpentine (10.1 wt%), plagioclase (other than albite, 8.3 wt%), albite (8.1 wt%), pyrrhotite (7.54 wt%) and muscovite ((KAl_2_(Si_3_Al)O_10_(OH, F)_2_), 5.6 wt%). The carbonate mineral content was small, and only 0.01 wt% of dolomite was detected. The total C concentration was 0.91% and the carbonate C concentration 0.11%. The sulphidic mineral content was 9.48 wt%, the sample containing 7.54 wt% of pyrrhotite, 1.11 wt% of pyrite, 0.82 wt% of chalcopyrite and 0.01 wt% of sphalerite. The sulphides appeared as single grains, primarily in the size fraction of 10–20 μm. The appearance of the sulphide grains varied, most being unweathered and some oxidised. The amount of detected oxidised Fe sulphides was 0.94 wt%. The total S concentration was 3.35% according to the Leco method and 3.72% according to calculation based on the sulphide mineral amounts. Based on the S and C concentrations, the Hitura 2014 sample was classified into group III.

The NAG pH of the Hitura 2014 sample was 2.6, and the NAG values were 22.1 kg H_2_SO_4_/t (pH 4.5) and 6.92 kg H_2_SO_4_/t (pH 7.0). The AP calculated based on the total S and applying the factor of 31.25 was 105 kg CaCO_3_/t (AP1) and 109 kg CaCO_3_/t with the factor of 62.5 (AP2). The minAP calculated based on minS was 67.3 kg CaCO_3_/t applying the factor of 31.25 (minAP1) and 135 kg CaCO_3_/t with the factor of 62.5 (minAP2). The NP according to the standard ABA test was 38.3 kg CaCO_3_/t, the carbNP calculated from the carbonate C concentration was 9.17 kg CaCO_3_/t and the minNP calculated by mineralogy was 24.8 kg CaCO_3_/t. Based on the calculations, the main three minNP-contributing minerals were biotite (8.99 kg CaCO_3_/t), olivine (8.31 kg CaCO_3_/t) and serpentine (6.70 kg CaCO_3_/t). The Hitura 2014 waste rock material was classified as acid-generating by all the applied ARD prediction methods.

In 2016, drainage water from the Hitura waste rock pile had a pH of 4.0, which was slightly less acid than in 2014. The alkalinity could not be measured. In the Hitura 2016 sample, the main minerals were biotite (25.4 wt%), plagioclase (other than albite, 18.0 wt%), albite (16.4 wt%), quartz (14.2 wt%) and muscovite (7.0 wt%). No carbonates were detected. The total C concentration was 1.04% and the carbonate C concentration < 0.05%. The sulphidic mineral content was 4.33 wt%, the sample containing 1.83 wt% of pyrrhotite, 0.10 wt% of pyrite, 0.07 wt% of chalcopyrite, 0.01 wt% of sphalerite and a mixture of sulphides and silicates (5.8 wt%), which was calculated as 2.32 wt% of pyrrhotite based on the SEM data. The sulphides appeared as single grains, pyrrhotite primarily in the size fraction of < 30 μm and pyrite in the size fraction of < 20 μm. The appearance of the sulphide grains varied, some being unweathered and some oxidised. The amount of detected oxidised Fe sulphides was 0.23 wt%. The total S concentration was 2.47% according to the Leco method and 1.63% according to calculation based on the sulphide mineral amounts. Based on the S and C concentrations, the Hitura 2016 sample was classified into group III.

The NAG pH of the Hitura 2016 sample was 2.6, and the NAG values were 25.1 kg H_2_SO_4_/t (pH 4.5) and 36.7 kg H_2_SO_4_/t (pH 7.0). The AP calculated based on the total S and applying the factor of 31.25 was 77.2 kg CaCO_3_/t (AP1) and 154 kg CaCO_3_/t with the factor of 62.5 (AP2). The minAP calculated based on minS was 26.5 kg CaCO_3_/t applying the factor of 31.25 (minAP1) and 53 kg CaCO_3_/t with the factor of 62.5 (minAP2). The NP according to the standard ABA test was 4.55 kg CaCO_3_/t, the carbNP calculated from the carbonate C concentration was 0 kg CaCO_3_/t and the minNP calculated by mineralogy was 13.6 kg CaCO_3_/t. Based on the calculations, the main minNP-contributing minerals were biotite (11.7 kg CaCO_3_/t) and almandine (1.9 kg CaCO_3_/t). The Hitura 2016 waste rock material was classified as acid-generating by most of the applied ARD prediction methods.

### Hammaslahti

The water pond below the Hammaslahti waste rock pile had an acid pH (3.9) and the alkalinity could not be measured. The main minerals were quartz (42.3 wt%), chlorite (8.3 wt%) and biotite (6.4 wt%). Some traces of gypsum were detected (silicate-gypsum mixture 0.02 wt%). The Hammaslahti sample contained a substantial amount of unclassified minerals (19.7 wt%). The carbonate mineral content was low, the sample containing 0.02 wt% of calcite. The total C concentration was 0.53% and the carbonate C concentration < 0.05%. The sulphidic mineral content was 0.97 wt%, the sample containing 0.83 wt% of pyrrhotite, 0.08 wt% of chalcopyrite and 0.06 wt% of pyrite. The sulphides appeared as single grains, primarily in the size fraction of 10–20 μm. The appearance of the sulphide grains varied, most being unweathered and some oxidised. The amount of detected oxidised Fe sulphides was 0.49 wt%. The total S concentration was 1.61% according to the Leco method and 0.37% according to calculation based on the sulphide mineral amounts. Based on the S and C concentrations, the Hammaslahti sample was classified into group III.

The NAG pH of the Hammaslahti sample was 3.0, and the NAG values were 8.35 kg H_2_SO_4_/t (pH 4.5) and 18.6 kg H_2_SO_4_/t (pH 7.0). The AP calculated based on the total S and applying the factor of 31.25 was 50.3 kg CaCO_3_/t (AP1) and 101 kg CaCO_3_/t with the factor of 62.5 (AP2). The minAP calculated based on minS was 6.32 kg CaCO_3_/t applying the factor of 31.25 (minAP1) and 12.6 kg CaCO_3_/t with the factor of 62.5 (minAP2). The NP according to the standard ABA test was 13.4 kg CaCO_3_/t, the carbNP calculated from the carbonate C concentration was 0 kg CaCO_3_/t and the minNP calculated by mineralogy was 6.97 kg CaCO_3_/t. Based on the calculations, the main three minNP-contributing minerals were biotite (2.95 kg CaCO_3_/t), chlorite (2.79 kg CaCO_3_/t) and Mg-hornblende (0.37 kg CaCO_3_/t). The Hammaslahti waste rock material was classified as acid-generating by most of the applied ARD prediction methods.

### Särkiniemi

Drainage water from the Särkiniemi waste rock pile had an acid pH (3.3) and the alkalinity could not be measured. The main minerals were biotite (33.6 wt%), plagioclase (other than albite, 19.7 wt%), quartz (16.6 wt%) and Fe-hornblende (6.1 wt%). No carbonates were detected. The total C concentration was 0.26% and the carbonate C concentration 0.05%. The sulphidic mineral content was 3.81 wt%, the sample containing 1.52 wt% of pyrrhotite, 0.03 wt% of pentlandite, 0.02 wt% of pyrite and a mixture of sulphides and silicates (2.54 wt%), which was calculated as 1.27 wt% of pyrrhotite based on the SEM data. The sulphides appeared as single grains, primarily in the size fraction of < 30 μm. The appearance of the sulphide grains varied, some being unweathered and some oxidised. The amount of detected oxidised Fe sulphides was 0.14 wt%. The total S concentration was 1.69% according to the Leco method and 1.07% according to calculation based on the sulphide mineral amounts. Based on the S and C concentrations, the Särkiniemi sample was classified into group III.

The NAG pH of the Särkiniemi sample was 2.7, and the NAG values were 18.4 kg H_2_SO_4_/t (pH 4.5) and 30.3 kg H_2_SO_4_/t (pH 7.0). The AP calculated based on the total S and applying the factor of 31.25 was 52.8 kg CaCO_3_/t (AP1) and 106 kg CaCO_3_/t with the factor of 62.5 (AP2). The minAP calculated based on minS was 16.9 kg CaCO_3_/t applying the factor of 31.25 (minAP1) and 33.8 kg CaCO_3_/t with the factor of 62.5 (minAP2). The NP according to the standard ABA test was 13.2 kg CaCO_3_/t, the carbNP calculated from the carbonate C concentration was 4.48 kg CaCO_3_/t and the minNP calculated by mineralogy was 19.6 kg CaCO_3_/t. Based on the calculations, the main three minNP-contributing minerals were biotite (15.5 kg CaCO_3_/t), serpentine (2.34 kg CaCO_3_/t) and Fe-hornblende (1.28 kg CaCO_3_/t). The Särkiniemi waste rock material was classified as acid-generating by most of the applied ARD prediction methods.

### Hällinmäki

Drainage water from the Hällinmäki waste rock pile had a neutral pH (6.7) and alkalinity of 8.4 mg/L CaCO_3_. The main minerals were plagioclase (other than albite, 42.2 wt%), Mg-hornblende (Ca_2_[Mg_4_(Al,Fe)]Si_7_AlO_22_(OH)_2_, 14.0 wt%), Fe-hornblende (8.4 wt%) and quartz (5.0 wt%). The carbonate mineral content was low, the sample containing 0.06 wt% of calcite. The total C concentration was < 0.05% and the carbonate C concentration < 0.05%. The sulphidic mineral content was 0.28 wt%, the sample containing 0.16 wt% of chalcopyrite, 0.11 wt% of pyrrhotite and 0.01 wt% of pyrite. The sulphides appeared as single grains and as inclusions inside silicates. The sulphide grains were unweathered and small, mainly just a few micrometres and 40 μm at maximum. No oxidized Fe sulphides were detected. The total S concentration was 0.37% according to the Leco method and 0.11% according to calculation based on the sulphide mineral amounts. Based on the S and C concentrations, the Hällinmäki sample was classified into group III.

The NAG pH of the Hällinmäki sample was 4.1, and the NAG values were 0.24 kg H_2_SO_4_/t (pH 4.5) and 2.63 kg H_2_SO_4_/t (pH 7.0). The AP calculated based on the total S and applying the factor of 31.25 was 11.5 kg CaCO_3_/t (AP1) and 22.9 kg CaCO_3_/t with the factor of 62.5 (AP2). The minAP calculated based on minS was 1.69 kg CaCO_3_/t applying the factor of 31.25 (minAP1) and 3.37 kg CaCO_3_/t with the factor of 62.5 (minAP2). The NP according to the standard ABA test was 9.52 kg CaCO_3_/t, the carbNP calculated from the carbonate C concentration was 0 kg CaCO_3_/t and the minNP calculated by mineralogy was 20.3 kg CaCO_3_/t. Based on the calculations, the main three minNP-contributing minerals were aegerine-augite (9.14 kg CaCO_3_/t), Mg-hornblende (3.42 kg CaCO_3_/t) and anorthite (2.95 kg CaCO_3_/t). The Hällinmäki waste rock material was classified as acid-generating by most of the applied ARD prediction methods, except when calculating the NPR as minNP/minAP.

### Laiva

Drainage water from the Laiva waste rock pile had a neutral pH (7.0) and alkalinity of 21.2 mg/L CaCO_3_. The main minerals were plagioclase (other than albite, 21.5 wt%), quartz (20.3 wt%), k-feldspar (13.8 wt%), biotite (10.9 wt%), albite (10.8 wt%), actinolite (Ca_2_(Mg,Fe)_5_Si_8_O_22_(OH)_2_, 7.9 wt%) and Fe-hornblende (5.9 wt%). No carbonates were detected. The total C concentration was < 0.05% and the carbonate C concentration < 0.05%. The sulphidic mineral content was low, the sample containing 0.10 wt% of pyrrhotite. The sulphides appeared as unweathered single grains. The sulphide grains were small, mainly just a few micrometres and 20 μm at maximum. No oxidised Fe sulphides were detected. The total S concentration was 0.10% according to the Leco method and 0.04% according to calculation based on the sulphide mineral amounts. Based on the S and C concentrations, the Laiva sample was classified into group III.

The NAG pH of the Laiva sample was 7.5, both NAG values (pH 4.5 and pH 7.0) being 0 kg H_2_SO_4_/t. The AP calculated based on the total S and applying the factor of 31.25 was 3.16 kg CaCO_3_/t (AP1) and 6.31 kg CaCO_3_/t with the factor of 62.5 (AP2). The minAP calculated based on minS was 0.06 kg CaCO_3_/t applying the factor of 31.25 (minAP1) and 0.12 kg CaCO_3_/t with the factor of 62.5 (minAP2). The NP according to the standard ABA test was 9.91 kg CaCO_3_/t, the carbNP calculated from the carbonate C concentration was 0 kg CaCO_3_/t and the minNP calculated by mineralogy was 8.70 kg CaCO_3_/t. Based on the calculations, the main three minNP-contributing minerals were biotite (5.01 kg CaCO_3_/t), actinolite (1.85 kg CaCO_3_/t) and Fe-hornblende (1.24 kg CaCO_3_/t). The applied ARD prediction methods gave conflicting ARD generation predictions for the Laiva waste rock material.

### Kevitsa

Drainage water from the Kevitsa waste rock pile had a neutral pH (7.4) and alkalinity of 100 mg/L CaCO_3_. The main minerals were diopside (47.9 wt%), tremolite (12.7 wt%) and serpentine (9.8 wt%). The carbonate mineral content was low, the sample containing 0.04 wt% of calcite. The total C concentration was 0.15% and the carbonate C concentration 0.1%. The sulphidic mineral content was 0.23 wt%, the sample containing 0.15 wt% of pyrrhotite, 0.05 wt% of pentlandite and 0.03 wt% of chalcopyrite. The sulphides appeared as unweathered single grains. The sulphide grains were small, mainly just a few micrometres and 40 μm at maximum. No oxidised Fe sulphides were detected. The total S concentration was 0.31% according to the Leco method and 0.09% according to calculation based on the sulphide mineral amounts. Based on the S and C concentrations, the Kevitsa sample was classified into group III.

The NAG pH of the Kevitsa sample was 9.1, both NAG values (pH 4.5 and pH 7.0) being 0 kg H_2_SO_4_/t. The AP calculated based on the total S and applying the factor of 31.25 was 9.56 kg CaCO_3_/t (AP1) and 19.1 kg CaCO_3_/t with the factor of 62.5 (AP2). The minAP calculated based on minS was 1.31 kg CaCO_3_/t applying the factor of 31.25 (minAP1) and 2.61 kg CaCO_3_/t with the factor of 62.5 (minAP2). The NP according to the standard ABA test was 57.7 kg CaCO_3_/t, the carbNP calculated from the carbonate C concentration was 8.1 kg CaCO_3_/t and the minNP calculated by mineralogy was 173 kg CaCO_3_/t. Based on the calculations, the main three minNP-contributing minerals were diopside (148 kg CaCO_3_/t), olivine (12 kg CaCO_3_/t) and serpentine (6.49 kg CaCO_3_/t). The Kevitsa waste rock material was classified as non-acid-generating by most of the applied ARD prediction methods, except when using the carbNP.

All samples contained some sulphides, which were mostly pyrrhotite and pyrite, with lesser amounts of chalcopyrite, pentlandite and sphalerite (Table 6). Most samples also contained some amounts of oxidised Fe sulphides transformed into sulphate-bearing Fe oxyhydroxides (or gypsum resulting from neutralisation), typically appearing around the healthy sulphides as a thin (a few micrometres) rim. The sulphides mainly appeared as single grains. The most common carbonates were calcite and dolomite, which were present in most carbonate-containing samples (Table 6). Magnesite was only present in both Horsmanaho samples and siderite in the Pampalo sample.

**Fig. 1:**
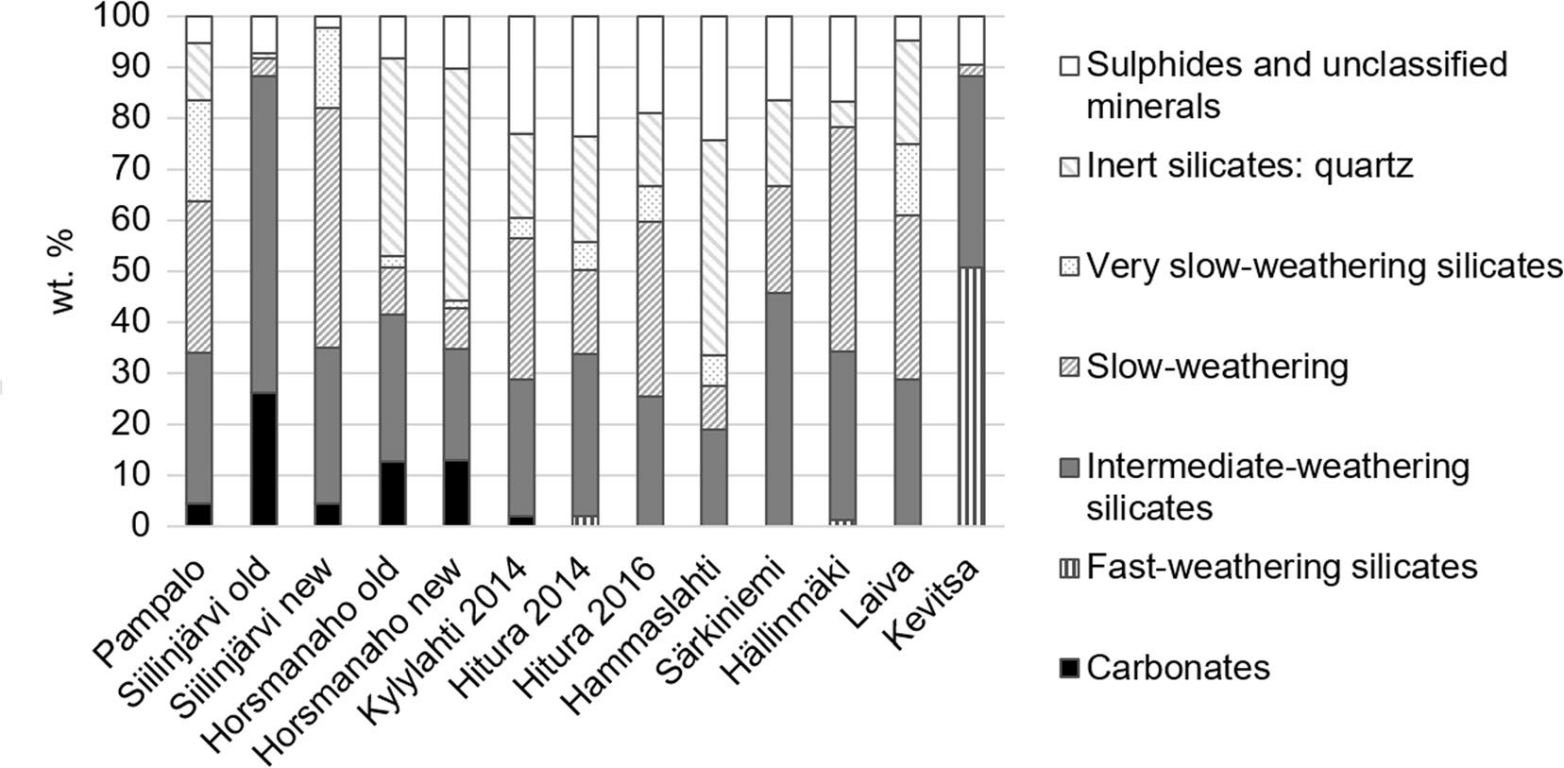
Mineral groups according to weathering intensity after Sverdup (1990), including a group of sulphides and unclassified minerals

Silicates of intermediate-weathering intensity were the dominant type in the waste rock samples (Fig. 1). These minerals consist of Mg and Fe silicates and Mg- or Fe-bearing aluminosilicates. Their content was greatest (> 40 wt%) in the samples Siilinjärvi old and Särkiniemi, respectively. The concentrations of the silicates of slow-weathering intensity were greatest (> 40 wt%) in the samples Siilinjärvi new and Hällinmäki, respectively. All samples contained very slowly weathering silicates at contents below 20 wt%, the highest amounts being detected in the samples Pampalo (19.7 wt%) and Siilinjärvi new (15.6 wt%).

**Fig. 2:**
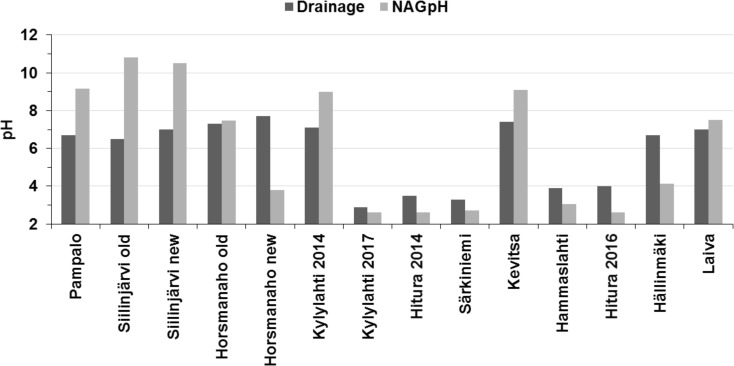
Comparison of the NAG pH and drainage pH values

The NAG pH was high (≥ 7.5) and the NAG value correspondingly zero in the samples of group I and group II, excluding the sample Horsmanaho new, and in the samples Kevitsa and Laiva of group III (Table 7). Group III had dominantly low NAG pH values (≤ 4.1). As presented in Fig. 2, the NAG pH was a pessimistic indicator in the cases of Horsmanaho new and Hällinmäki samples and over-optimistic in the case of group I samples.

**Fig. 3:**
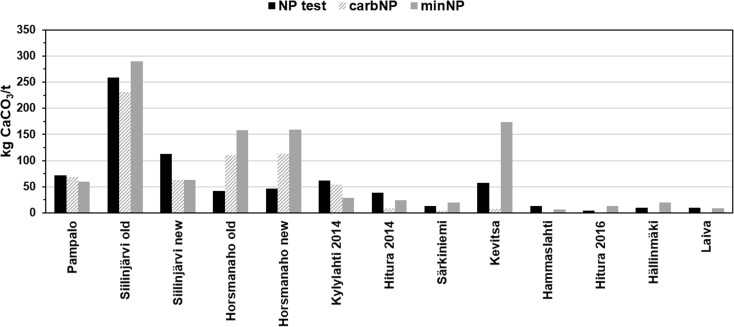
Comparison of NP values obtained using different methods

A comparison of NP values obtained by different methods is presented in Fig. 3. The EN 15875 ABA test NP was highest in the Pampalo and Siilinjärvi new samples of group I, in the Kylylahti 2014 sample of group II and in the Hitura 2014, Hammaslahti and Laiva samples of group III. The mineralogical NP was highest in the Siilinjärvi old sample of group I, in both Horsmanaho samples of group II, and in the Särkiniemi, Kevitsa, Hitura 2016 and Hällinmäki samples of group III. The carbonate NP was usually the lowest of the different NP values, excluding the Pampalo sample of group I, in which minNP was lower, and group II samples, in which the ABA NP values of the both Horsmanaho samples were lower and the minNP of the Kylylahti 2014 sample was lower. In general, the smallest differences between NP values were observed in the group I samples (Fig. 3).

Based on the results presented in Table 8, carbonates are the main NP contributors in the group I samples of Pampalo (most NP from dolomite), Siilinjärvi old (dolomite) and Siilinjärvi new (calcite) and in the group II sample of Kylylahti 2014 (calcite). Silicate minerals dominate the NP potential of the other samples, the main NP contributor being biotite in Hitura 2014 and 2016, Hammaslahti, Särkiniemi and Laiva. Diopside was the most important NP mineral in the Kevitsa sample and aegerine-augite in the Hällinmäki sample.

**Fig. 4:**
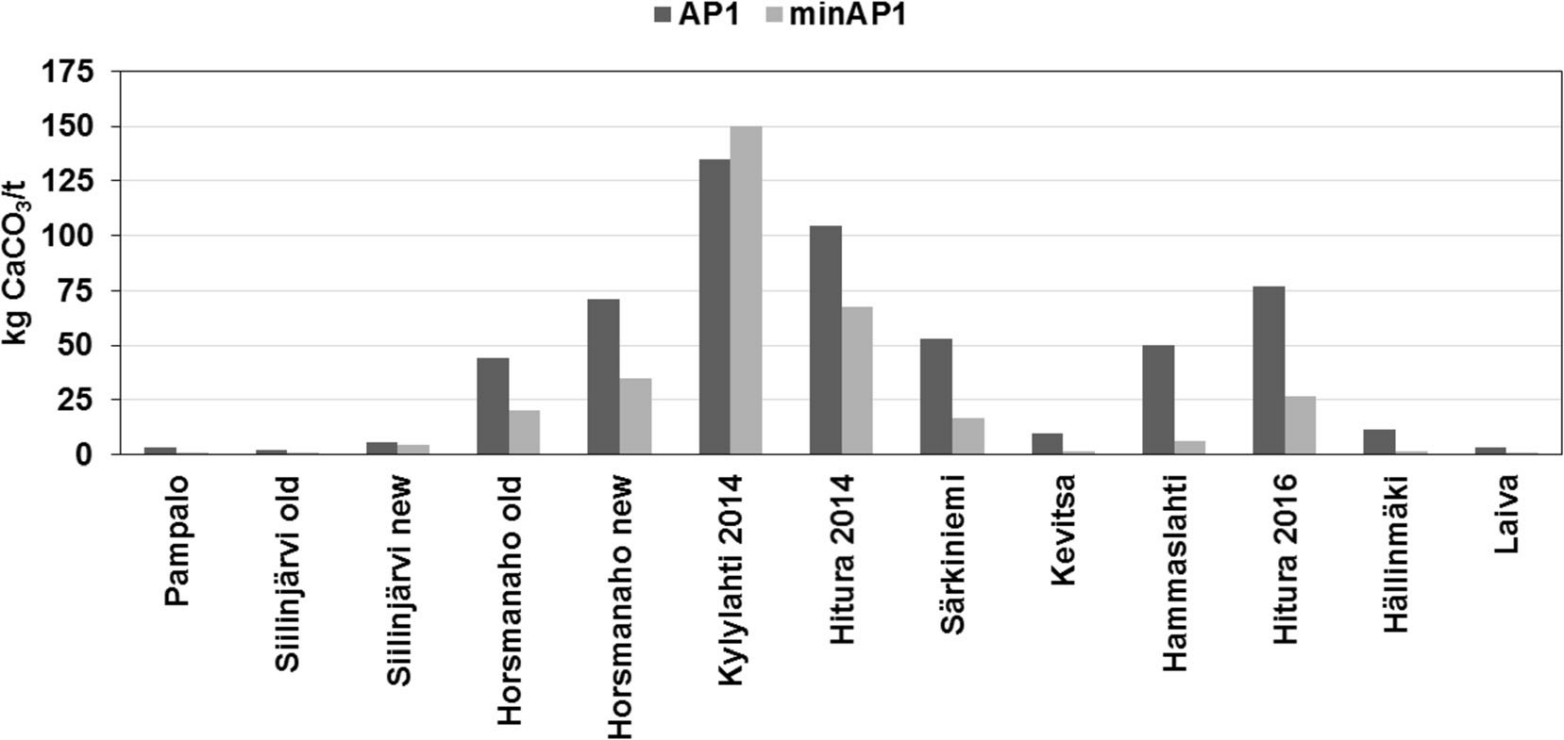
Comparison of AP values obtained using the EN 15875 ABA test and mineralogical calculation

Sulphur concentrations obtained using the Leco method were notably higher in the majority of the samples than the S concentrations calculated based on mineralogy (Table 7). The minS was higher in two samples, Kylylahti 2014 and Hitura 2014, which had the highest S concentrations in general (Leco S 4.32 and 3.35% and minS 4.97 and 3.72%, respectively). A comparison of AP values obtained using the EN 15875 ABA test and mineralogical calculations using the factor of 31.25 is presented in Fig. 4. EN ABA test AP1 values are higher in all samples, excluding the Kylylahti 2014 sample of group II. In general, the smallest differences between AP1 and minAP1 samples were observed in the group I samples (Fig. 4).

According to the ABA results (NP/AP ratios), samples of group I (Pampalo, Siilinjärvi old and new) consist of non-acid-generating waste rocks (Tables 9 and 10), whereas the other waste rocks (groups II and III) are acid-generating, except for the Kevitsa and Laiva waste rocks. Based on NAG pH and NAG values, the rock samples of the first group were non-acid-generating, similarly to the determination based on the ABA test. Congruently with the ABA test, the NAG test classified the Horsmanaho new sample from group II and the Hitura 2014 and Särkiniemi samples from group III as acid-generating. In contrast to the ABA test, the NAG test classified the Horsmanaho old and Kylylahti 2014 samples from group II and Kevitsa and Laiva samples from group III as non-acid-generating.

## Discussion

This study focused on 14 waste rock and 14 corresponding seepage water samples from 10 Finnish mine sites where thorough static testing was undertaken. The investigated static tests were compared with each other and with the corresponding seepage pHs. All the static tests have known limitations related, for instance, to the rock texture, mineral surface area, mineral assemblage and mineral impurities that might affect the weathering rate, small sample sizes and crushing of the samples (White III et al. 1999; Paktunc 1999b; Jambor 2003; Parbhakar-Fox and Lottermoser 2015; Dold 2017). Compared to this study, in which pulverised rock samples were used and valuable information on the rock texture was mainly lost, the mineralogical prediction accuracy can be further enhanced with a textural investigation and assessment of the intact rocks (Parbhakar-Fox et al. 2011; Brough et al. 2013). For this purpose, the acid rock drainage index (ARDI) has been developed, as part of the geochemistry-mineralogy-texture (GMT) approach (Parbhakar-Fox et al. 2011).

For both the commonly used static tests and the calculations based on mineralogy, the key element in successful characterisation is representative sampling (Price 2009). For this study, thorough sampling of the waste rock piles was not possible and only composite surface samples were collected. The known limitations should be taken into account when inspecting the geochemical rock analysis results and drainage quality data. Correct environmental sampling techniques are presented, for example, by McLemore et al. (2014).

It should be emphasized that while the static tests and mineralogical calculations are suitable for preliminary screening in the ARD assessment of mine waste, more detailed and site-specific investigations should still be carried out, not to exclude investigations to predict metal-rich neutral mine drainage (NMD, Dold 2017). As this study concentrated on preliminary screening purposes, assuming sulphide oxidation by oxygen, more complicated further reactions such as oxidation by Fe^3+^ (Dold 2010) and the precipitation of Fe^3+^ and Al^3+^ hydroxides (Stumm and Morgan 1996; Lottermoser 2010) should be taken into account when performing detailed site-specific investigations.

According to the results, ARD prediction based on SEM mineralogy and mineralogical NP calculation appears to be an accurate tool compared to the common static laboratory methods. The mineralogical ARD calculation could, for instance, be incorporated in mineralogical analyses and the results regarded to be as adequate as the results from the commonly used static tests. The weathering groups of minerals according to Sverdrup (1990) might be considered to be too broad for ARD prediction. For example, pyroxene, amphibole, biotite and chlorite are all grouped as intermediate-weathering with the same relative reactivity value (Jambor and Blowes 1998). Nevertheless, mineralogical investigation reveals information about the actual minerals corresponding to the AP and NP, which, besides the calculation of AP and NP values, also permits the assessment of reaction rates of significant minerals compared to each other. This study highlighted the need for further investigation into the contribution of silicates to the neutralisation capacity in calcite-poor environments and especially into the ability of silicates to react to potentially rapid acid surges generated by pyrrhotite weathering.

### Determination of AP

The Fe sulphides pyrrhotite and pyrite, respectively, appear to be the most widely occurring sulphide species in Finnish waste rocks, also being responsible for the majority of acid production. Pyrrhotite was calculated to be the main AP-producing mineral at 6/10 of the mine sites. This is contradictory to the common assumption that pyrite is the greatest contributor to ARD (Nordstrom and Alpers 1999). As the common static ABA tests do not provide information on the S minerals, as pointed out by Dold (2017), the oxidation of different sulphides produces fewer protons than pyrite, and the main problem of the EN 15875 ABA tests seems to be the overestimation of the AP. Besides the different numbers of protons released, sulphide species also have different resistances to oxidation. According to Jambor (1994), the oxidation resistance from least to highest is pyrrhotite ➝ sphalerite/galena ➝ pyrite-arsenopyrite ➝ chalcopyrite ➝ magnetite. Thus, as pointed out by Jambor and Blowes (1998), although pyrite can theoretically generate more acid than pyrrhotite, the slower weathering rate of pyrite, and hence the lower capacity to overwhelm neutralisation reactions, introduces uncertainties in predictions based on static ABA tests. The oxidation resistance order of different sulphide species might change when factors such as biological (Kuyucak 2002) and galvanic (Kwong et al. 2003) interactions are considered. The stability of individual sulphides is also related to the interstitial impurities and trace elements, which usually render sulphides more susceptible to weathering (Kwong 1993).

As pyrrhotite appears to be the most important acid producer at Finnish mine sites, more effort should be directed to studies related to the occurrence of different pyrrhotite types and the ability of silicates to resist a possible rapid pyrrhotite-originated acid surge. The reaction rates of pyrrhotite have been noted to be up to 100 times faster than those of pyrite at 25 °C and in atmospheric oxygen (Nicholson 1994; Nicholson and Scharer 1994). The main factor for higher oxidation rate is the higher surface area of pyrrhotite compared with the other sulphides, e.g. pyrite (Janzen et al. 2000). Although in general the oxidation of pyrrhotite is relatively fast, the structural differences can cause variable dissolution behaviour of pyrrhotite (Thomas et al. 1998).

When inspecting uncertain cases, e.g. samples from Hällinmäki and Särkiniemi, the uncertainties related to ARD prediction by static methods could be reduced by examining the sulphide species. In many Finnish waste rock facilities, the neutralisation capacity is related to silicate minerals, and fast-reacting carbonates are absent. If the main AP contributor in the Nordic climate is slowly weathering sulphide, such as chalcopyrite in Hällinmäki, this suggests that the silicate-based NP could respond to the acidity, as also appears to be the case when observing the actual drainage quality at the Hällinmäki mine site. On the other hand, if the main AP contributor is fast-oxidising pyrrhotite, it can produce a surge of acidity that will overwhelm the immediately available neutralising capacity of associated silicates, as can be observed at the Särkiniemi mine site.

The AP is often calculated based on the total S concentration, which has been debated by White et al. (1999) and Parbhakar-Fox et al. (2011), among others. The main complication is related to the fact that non-acid-forming S-bearing minerals, such as gypsum and barite, are included in the analysis and therefore in the AP potential. According to the mineralogical investigations in this study, barite does not appear to be a very common mineral in Finnish waste rock material, although some well-known exceptions exist, e.g. the barite-bearing Pyhäsalmi (Mäki et al. 2015) and Pahtavaara (Korkiakoski and Kilpelä 1997) deposits. Small amounts of gypsum and silicate-gypsum mixture were detected at 5/10 mine sites. The detected amounts were small, between 0.01 and 0.02 wt%, but considering that some gypsum probably remained undetected, gypsum might be a critical contributor to the total S and therefore the AP, increasing uncertainty in some ARD assessment cases. In these cases, pre-treating samples to remove gypsum should be considered. For example, samples can be digested with sodium carbonate to remove sulphate minerals (Lapakko 2002).

As the AP values obtained for static ABA testing are usually already overestimated, as discussed above, the use of the factor 62.5 instead of the commonly used 31.25 results in even more pessimistic ARD predictions (Tables 9 and 10). On the other hand, the use of the higher factor is well justified in cases when bicarbonate is the main product of carbonate dissolution (Dold 2017). In addition, the ARD predictions based on mineralogically calculated minNP and minAP obtained with the factor of 62.5 (Table 10) appear to result in realistic predictions compared to the actual drainage qualities.

### Determination of the NP

According to the calculated NP values, silicates appear to be an important contributor to ARD prevention at Finnish mine sites. Carbonates are the main source of NP at 4/10 of the investigated mine sites, of which calcite is the main source of NP at only two sites. At the remaining 6/10 of the investigated mine sites, silicate minerals are the most important contributors to the NP. Of the silicate minerals, biotite was observed to be the most important contributor to the neutralisation capacity at 4/10 of the investigated mine sites, followed by the other silicate minerals of the fast- and intermediate-weathering groups. Chlorite and mica have also been demonstrated to have a long-term acid-neutralising capacity in previous studies (Becker et al. 2015). As carbonates appear to be less common in Finnish mine wastes, the application of carbNP (NP calculated from the carbonate C concentration) is not widely recommendable. On the other hand, carbNP can be used in ARD predictions for mine wastes in which carbonates are slowly weatherable (e.g. magnesite) and therefore weakly leachable in the NP test and in which carbonates are main contributors to the neutralisation capacity.

**Fig. 5:**
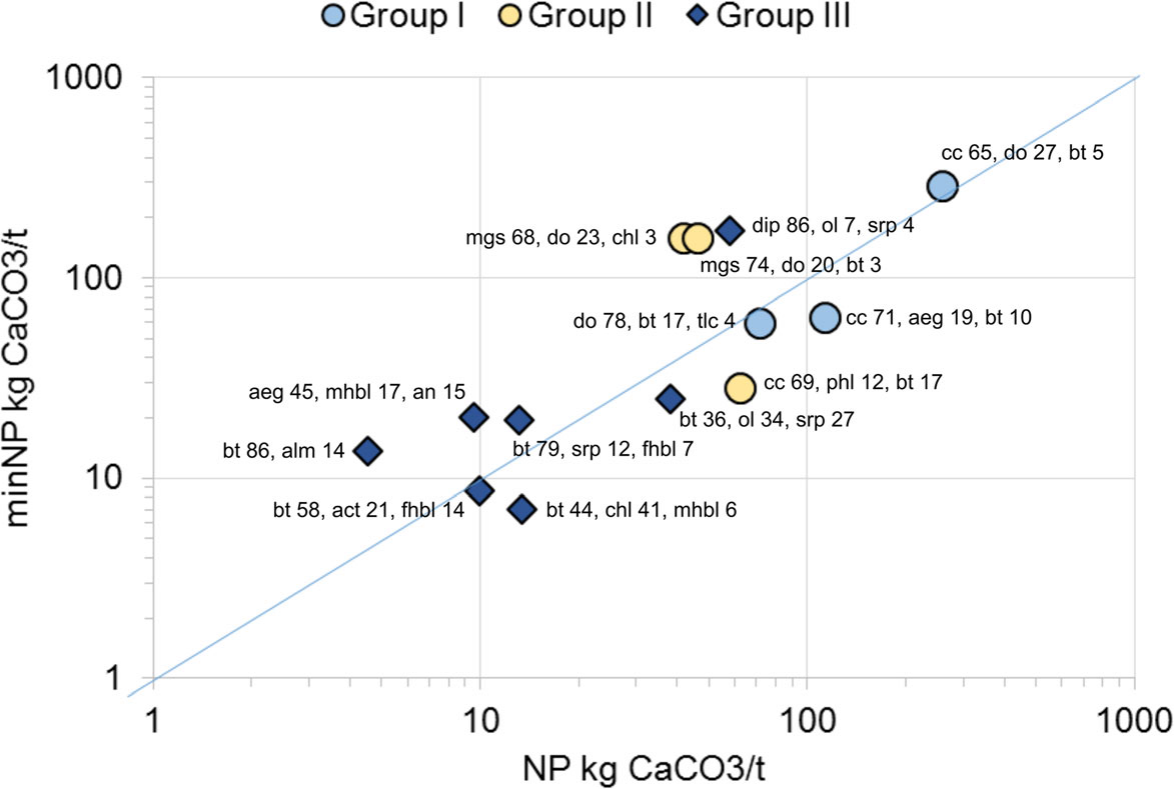
Comparison of the mineralogical NP with the measured NP values and the three most significant minNP-contributing minerals and percentage contribution of the minerals to the total calculated minNP. Abbreviations for the minerals: act = actinolite, aeg = aegerine-augite, alm = almandine, an = anorthite, bt = biotite, cc = calcite, chl = chlorite, dip = diopside, do = dolomite, fhbl = Fe-hornblende, mgs = magnesite, mhbl = Mg-hornblende, ol = olivine, phl = phlogopite, srp = serpentine, tlc = talc

Differences between the NP measured using the standard EN 15875 method and minNP calculated from the mineral content can be observed in Fig. 5. Magnesite appears to be considerably underestimated in the measured NP. According to the dissolution studies by Hoşgün and Kurama (2012), magnesite should mostly dissolve in 1 M HCl solution. On the other hand, the results of this study suggest that most of the magnesite did not dissolve in the 1 M HCl used in the NP measurement, and magnesite has also been noted to react slowly in laboratory ABA tests by Paktunc (1999b). Based on Fig. 5, other minerals that might be undervalued by the laboratory measured NP (i.e. not well leached in HCl) are diopside, aegerine-augite, hornblende, anorthite and biotite. In some cases, the higher minNP values could also result from the fact that the mineralogical calculations were based on the relative reactivity values determined in slightly acid conditions (pH 5.0; Sverdrup 1990), whereas in most of the investigated mine waste sites, the pH is close to neutral. Therefore, the minNP values might overestimate the neutralising capacity, as the overall mineral dissolution is lower at a higher pH (Heikkinen and Räisänen 2008).

Apparently, the measured NP might slightly overestimate the neutralisation capacity of olivine, which has also been noted by Heikkinen and Räisänen (2008). This might be due to the HCl used in the EN ABA test, which dissolves some silicates more efficiently than is assumed in minNP calculations (Heikkinen and Räisänen 2008). Acids have been noted to more readily dissolve olivine compared to various other minerals, e.g. pyroxenes and some phyllosilicates (Terry 1983). Underestimation of biotite (due to its abundance) in the NP based on titration can also be noted in the Hitura 2016 sample.

The behaviour and dissolution of minerals in static laboratory tests and field sites are complicated and affected by several factors (Mase 1961; Prosser 1970; Currel et al. 1972; Sanemasa et al. 1972; Terry 1983; Nagy 1995; Critelli et al. 2014). The dissolution of significant NP-contributing minerals should be more thoroughly investigated, especially regarding common ARD prediction methods, as according to the results, the EN ABA test and NAG test do not correctly consider certain mineral types. In general, as silicate minerals appear to have significant importance for the neutralisation capacity and therefore for ARD prediction, the relative reactivities and dissolution rates of these minerals should be more thoroughly investigated in acids and mine waste environments and taken into greater account in NP assessments.

### ARD prediction based on the NAG test

When using the traditional NAG pH criterion of pH ≤ 4.5 for potentially acid-generating rock material, the NAG test pH was observed to be a pessimistic indicator in some cases. In the Horsmanaho new sample, the main AP contributor was a high amount (3.59 wt%) of fast-reacting pyrrhotite, whereas the main NP contributor was magnesite, which has also been noted to react slowly in laboratory ABA tests by Paktunc (1999b). The relatively low NAG pH of the Hällinmäki sample suggests that otherwise slowly weathering chalcopyrite is oxidised by the hydrogen peroxide, whereas the neutralisation capacity of the silicates could not be used. If the NAG pH, with the criterion of a pH between 3.21 and 4.52, was used for the uncertainty zone (UC), as proposed by Oh et al. (2017), Horsmanaho new and Hällinmäki samples would be classified as UC instead of PAF. It should be noted that when the NAG pH is lower than 4.5, the short-term risk of acid release is high, as a less acid-neutralising capacity might be readily available (Oh et al. 2017).

The results suggest that the weathering state of the sample has a significant impact on the NAG test performance. As was suspected based on the AMIRA guidebook (Smart et al. 2002), the most basic single-addition NAG test was not sufficient for the Kylylahti 2014 sample, which contained a high amount (9.53 wt%) of sulphides. Apparently, the added hydrogen peroxide was depleted before all the reactive sulphides were oxidised, as incomplete oxidation of sulphides can already appear in samples containing < 0.7–1 wt% pyritic sulphide (Stewart 2005). For samples containing high amounts of sulphides, the AMIRA guidebook (Smart et al. 2002) recommends the use of sequential NAG tests, where successive additions of peroxide are used. The Kylylahti 2014 sample was also relatively unweathered and fresh, which might affect the efficiency of hydrogen peroxide. The single-addition NAG test of the Kylylahti 2017 rock sample, which was similar to the Kylylahti 2014 sample but more weathered, resulted in more realistic NAG values.

The chemical mechanisms that control the stability and behaviour of the hydrogen peroxide solution are still insufficiently understood (Charles et al. 2015). Therefore, further investigation of the NAG test’s suitability for different rock types is needed. For example, the slowly reacting buffering minerals might require a longer time to react with hydrogen peroxide than overnight, which is specified in the AMIRA guidebook (Smart et al. 2002). Nevertheless, the NAG pH can be considered as a plausible single indicator in ARD assessment, as the measurement is simple and applicable to various samples (Oh et al. 2017). To enhance the usability of the NAG test, Parbhakar-Fox et al. (2018) highlight the importance of understanding the mineralogy of the sample prior to testing and developing a site-specific NAG testing protocol prior to new extractive waste classification projects. Furthermore, to reduce the discrepancies between laboratory results, standard experiment protocols and standard reference materials should be developed and used (Parbhakar-Fox et al. 2018).

## Conclusions

At Finnish mine waste sites, pyrrhotite appears to more commonly occur than pyrite. Pyrrhotite is also responsible for the main part of acid production. The standard ABA test results in too pessimistic ARD predictions, as the AP is overestimated by assuming that all S is pyritic.

At most of the investigated mine sites, silicate minerals were found to be the most important contributors to the neutralisation potential. Of the silicate minerals, biotite was observed to be the most important contributor to the total NP, followed by the other silicate minerals of the fast- and intermediate-weathering groups. As the carbonates appear to be less important NP producers in Finnish mine wastes, the application of carbNP is not widely recommendable. For the NAG test, the slowly reacting buffering minerals might require a longer time to react with hydrogen peroxide than overnight, which is specified in the AMIRA guidebook.

As silicate minerals appear to have significant importance for the neutralisation capacity and therefore ARD prediction at Finnish mine waste sites, the behaviour, relative reactivities and dissolution rates of these minerals should be more thoroughly investigated. More effort should especially be directed to studies related to the ability of silicates to resist the potentially rapid acid surges originating from pyrrhotite oxidation.

SEM mineralogy-based AP and NP calculation appears to be an efficient tool for ARD prediction. As an enhancement to the common static laboratory tests, mineralogical investigation reveals the actual minerals corresponding to the AP and NP, also permitting assessment of the reaction rates of significant minerals compared to each other. If sufficient suitable mineralogical data are available, there is less need to perform static ARD tests, which reduces analytical costs. Instead, funding could be used to investigate the possibility of NMD or the mobility of potentially harmful elements, as well as for more detailed kinetic testing of waste materials.
